# Experience-dependent, sexually dimorphic synaptic connectivity defined by sex-specific cadherin expression

**DOI:** 10.1126/sciadv.adq9183

**Published:** 2024-11-13

**Authors:** Chien-Po Liao, Maryam Majeed, Oliver Hobert

**Affiliations:** Department of Biological Sciences, Columbia University, Howard Hughes Medical Institute, New York, NY 10027, USA.

## Abstract

Early-life experience influences subsequent maturation and function of the adult brain, sometimes even in a sex-specific manner, but underlying molecular mechanisms are poorly understood. We describe here how juvenile experience defines sexually dimorphic synaptic connectivity in the adult *Caenorhabditis elegans* nervous system. Starvation of juvenile males disrupts serotonin-dependent activation of the CREB transcription factor in a nociceptive sensory neuron, PHB. CREB acts through a cascade of transcription factors to control expression of an atypical cadherin protein, FMI-1/Flamingo/CELSR. During postembryonic development, FMI-1 promotes and maintains synaptic connectivity of PHB to a command interneuron, AVA, in both sexes, but a serotonin-dependent transcriptional regulatory cassette antagonizes FMI-1 expression in males, thereby establishing sexually dimorphic connectivity between PHB and AVA. A critical regulatory node is the CREB-target LIN-29, a Zn finger transcription factor that integrates four layers of information: sexual specificity, past experience, time and cell-type specificity. Our findings provide the mechanistic details of how an early juvenile experience defines sexually dimorphic synaptic connectivity.

## Teaser

A transcriptional cascade shapes experience-dependent, sexually dimorphic synaptic connectivity in the brain.

## INTRODUCTION

Early-life experiences, such as stress and depression, can affect later developmental processes and can also affect the manifestation of neurological disorders (*1*, *2*). Notably, these past experiences can generate outcomes in a sex-dependent manner. For example, clinical studies indicate that females have a higher chance of developing anxiety or depression when experiencing early-life adversity (*3*). However, how past experiences influence later aspects of nervous system function in a sex-dependent manner remains elusive in humans. In rodents, a recent study has shown that juvenile adversity alters corticolimbic connectivity in traumatized female but not male rats, and, consequently, female rats are more likely to display depression behavior (*4*). Here, again, the molecular mechanisms that drive such sexually dimorphic outcomes are unknown.

Using the nematode *Caenorhabditis elegans* as a model, we have previously shown that the experience of early juvenile starvation affects the establishment of sexually dimorphic synaptic connectivity during sexual maturation (*5*). Specifically, the phasmid sensory neuron PHB generates en passant synapses onto the command interneuron AVA in both sexes at early juvenile stages; however, upon sexual maturation, this synaptic connection is sex-specifically maintained and progressively strengthened in hermaphrodites but eliminated in males (*5*–*9*). Early starvation increases the expression of the invertebrate norepinephrine analog octopamine to inhibit serotonin release from a pair of head sensory neurons (*5*). This transient serotonin depletion and consequent lack of activation of the metabotropic SER-4 receptor in PHB results in the failure of the PHB>AVA synaptic pruning and instead promotes growth of PHB>AVA synaptic connectivity in males, thereby eliminating the sexually dimorphic nature of such connectivity (*5*).

Serotonin-mediated activation of metabotropic receptors can affect a wide range of signaling pathways (*10*). We show here that the relevant readout in sex-specific serotonin-mediated synaptic pruning is a multilayered transcriptional response, in which serotonin signaling first activates the cAMP (adenosine 3′,5′-monophosphate) response element–binding protein (CREB) transcription factor, a phylogenetic conserved effector of neuronal plasticity (*11*). We discovered that CREB directly activates the Zn finger transcription factor LIN-29A, a key regulatory node in this process. Transcription of the *lin-29a* locus bookmarks feeding status via CREB activation, which cooperates with a cell-type–specific terminal selector to direct *lin-29* expression to specific neuron types. Activation of *lin-29a* transcription is antagonized in hermaphrodites by the TRA-1 master regulatory of sexual identity. *lin-29a* transcripts are translationally inhibited by the LIN-41 RNA binding protein until sexual maturation. Once LIN-29A protein is produced at the right place and time, it directs sexually dimorphic PHB>AVA connectivity by repression of another transcription factor, the Doublesex transcription factor, DMD-4, which we had previously found to be expressed in PHB of hermaphrodites but not of males (*12*). We identify the nonconventional cadherin *fmi-1/Flamingo* as the terminal effector of the CREB>LIN-29A>DMD-4 transcription factor cascade. We show that FMI-1 is normally required in hermaphrodites to promote the increase in the number of PHB>AVA en passant synapses. Therefore, male-specific repression of FMI-1 via serotonin/CREB-mediated LIN-29A induction (and DMD-4 repression) leads to a failure to sustain and expand PHB>AVA synapse number. Hence, we have discovered a mechanism whereby food perception in early life is translated into the sculpting of a sexually dimorphic synaptic connection later in development, during sexual maturation.

## RESULTS

### Sexually dimorphic PHB>AVA connectivity and neurite contact length is visualized with reporter gene technology

**Fig. 1.**
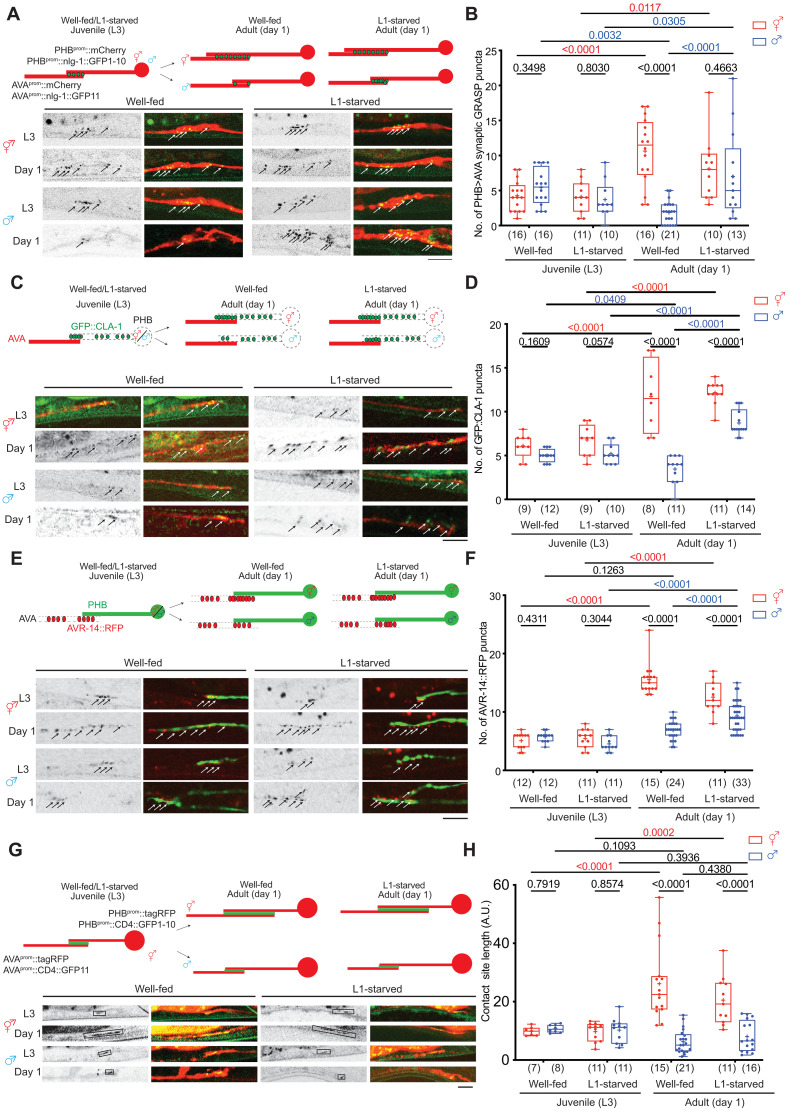
Juvenile serotonin signaling patterns sexually dimorphic synaptic connectivity. (**A** and **B**) Representative images (A) and quantification (B) of PHB>AVA synaptic GRASP(*otIs839*) in L3 and day 1 well-fed and L1-starved animals in both sexes. (**C**) Schematic illustration (top) and representative images (bottom) of AVA-juxtaposed GFP::CLA-1 in PHB (*otIs883;otEx8040*) in L3 and day 1 well-fed and L1-starved animals in both sexes. (**D**) Quantification of AVA-juxtaposed GFP::CLA-1 in PHB in L3 and day 1 well-fed and L1-starved animals in both sexes. Note for (C) and (D) that the number of AVA-juxtaposed CLA-1 puncta remained sexually dimorphic in the L1-starved adult male, which is consistent with electron micrographic data that show that PHB generates many sex-specific synapses, in addition to AVA (*9*). (**E**) Schematic diagram (top) and representative images (bottom) of PHB-juxtaposed AVR-14::TagRFP in in L3 and day 1 well-fed and L1-starved animals in both sexes. (**F**) Quantification of AVR-14::TagRFP in AVA [*otIs902; him-8(e1489)*] in L3 and day 1 well-fed and L1-starved animals in both sexes. (**G**) Schematic diagram (top) and representative images (bottom) of PHB>AVA neurite CD4-GRASP(*otEx8152*) in L3 and day 1 well-fed and L1-starved animals in both sexes. We measure the GFP-positive length to indicate the PHB/AVA contact site. (**H**) Quantification of CD4-GRASP(*otEx8152*) in L3 and day 1 well-fed and L1-starved animals in both sexes. Statistics: [(B), (D), (F), and (H)] Two-way analysis of variance (ANOVA) followed by Bonferroni multiple comparisons test. *P* value and *n* numbers are indicated on the graph. + indicates the mean value. Scale bars, 10 μm. A.U., arbitrary units.

Juvenile starvation (starving animals at the L1 stage for 24 hours and transferring them back to food) affects sexually dimorphic of PHB>AVA synapse number during sexual maturation via serotonin [5-hydroxytryptamine (5-HT)] signaling in the sensory neuron PHB (*5*). The molecular pathway underlying such experience-dependent, sex-specific synaptogenesis remains unknown. We had previously visualized the effect of feeding state and 5-HT signaling on PHB>AVA synaptic connectivity through the use of “GFP-reconstitution across synaptic partner” (GRASP) technology that exploits the synaptically localized neuroligin protein NLG-1 (*5*, *13*). We validated and expanded our previous results through the establishment and usage of additional reagents. First, an independently generated PHB>AVA GRASP reporter transgene, *otIs839*, confirmed our previous results in experience-dependent sexual dimorphic connectivity of PHB and AVA (Fig. 1, A and B) (*5*). Second, we confirmed that NLG-1–based GRASP is a proper indicator of PHB>AVA synaptic connectivity by using a green fluorescent protein (GFP)–tagged synaptic active zone marker, CLA-1/Clarinet, expressed specifically in PHB, as well as a postsynaptic marker, the ionotropic glutamate receptor AVR-14 that is expressed in the AVA neurons, the postsynaptic target of the glutamatergic PHB neurons (*14*, *15*). While GFP::CLA-1 alone labels all synaptic outputs of PHB, including those to many male-specific neurons, adjacent localization of PHB-expressed GFP::CLA-1 and AVA-expressed AVR-14::TagRFP signals represents an indicator of PHB>AVA connectivity (Fig. 1, C to F). We found that either in isolation or in combination, the number of GFP::CLA-1 and AVR-14::TagRFP signals corroborate the conclusions based on the GRASP constructs: The number of en passant PHB>AVA synaptic signals (i) show no dimorphisms in juvenile stages, (ii) display dimorphisms after sexual maturation, and (iii) increase in response to juvenile starvation in males (Fig. 1, E and F, and fig. S1, A and B).

Our previous global analysis of neurite adjacency and en passant synapse formation throughout the *C. elegans* nervous system indicated that the extent of adjacency of two neurites can be a sufficient predictor of the number of synapses formed between adjacent neurons (*16*). Because the PHB>AVA synapses are generated en passant along the PHB and AVA neurites, we considered the possibility that the extent of neurite adjacency is also sexually dimorphic and regulated by juvenile experience. We visualized PHB/AVA neurite contact length by using the transmembrane CD4 protein to direct the two halves of GFP (GFP_1-10_ and GFP_11_) to the surface of the PHB and AVA membranes, respectively. As previously demonstrated in other *C. elegans* cell types (*13*), GFP should only reconstitute when the two neurite membranes contact each other. We measured reconstituted GFP-positive neurite length to indicate the length of the PHB/AVA contact site (Fig. 1G). We found that, in juvenile animals, the PHB/AVA contact length was comparable between both sexes; however, in day 1 adults, the contact length was significantly increased in hermaphrodites but not in males (Fig. 1, G and H). Unexpectedly, while juvenile starvation affects the manifestation of sexually dimorphic synapse number, it did not affect sexually dimorphic neurite contact length (Fig. 1, G and H). Hence, the extent of sexually dimorphic neurite contact and sexually dimorphic synapse number can be uncoupled. Below, we define a molecular pathway that is dedicated toward controlling sexually dimorphic synaptogenesis, without affecting the sexually dimorphic extent of neurite contact.

### CRH-1/CREB functions downstream of juvenile serotonin signals to control male-specific synaptic remodeling upon sexual maturation

**Fig. 2.**
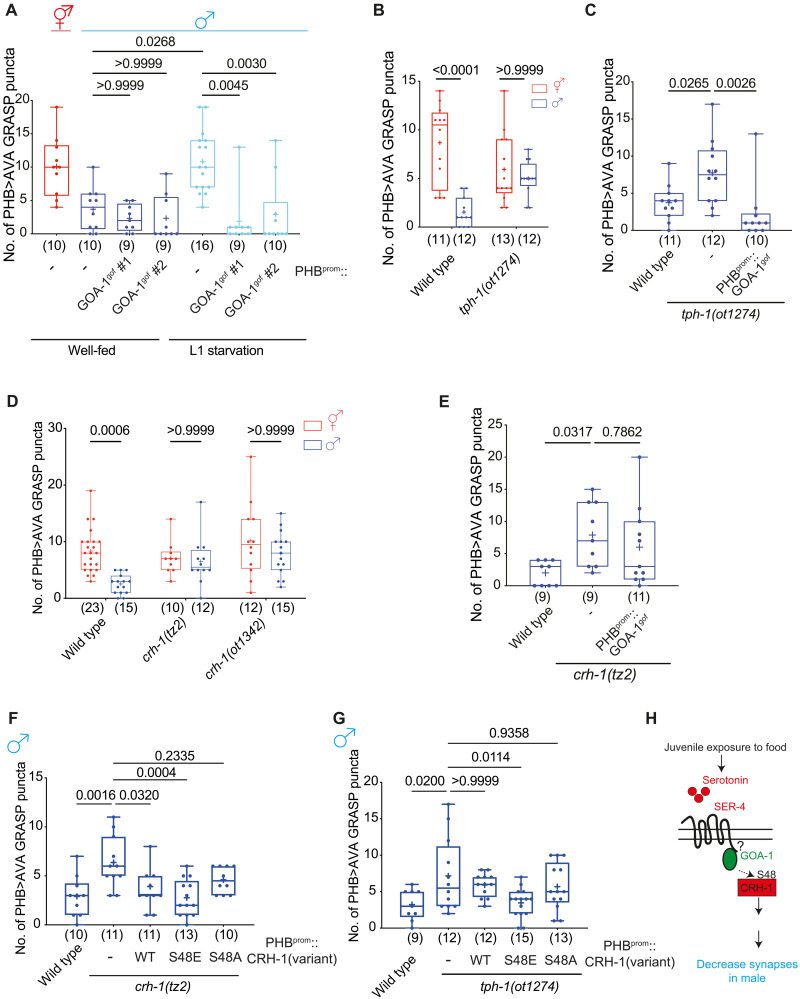
Juvenile starvation controls sexually dimorphic PHB>AVA synaptic contacts via CRH-1/CREB. (**A**) Quantification of PHB>AVA synaptic GRASP(*otIs839*) in well-fed wild-type animals and L1-starved wild-type and animals expressing PHB::GOA-1^gof^(*otEx7925* and *otEx8158*). (**B**) Quantification of PHB>AVA synaptic GRASP(*otIs839*) in wild type and *tph-1(ot1274)* in both sexes. (**C**) Quantification of PHB>AVA synaptic GRASP(*otIs839*) in wild-type and *tph-1(ot1274)* males expressing PHB::GOA-1^gof^(*otEx7925* and *otEx8158*). (**D**) Quantification of PHB>AVA synaptic GRASP(*otIs839*) in wild-type and *crh-1(tz2)* and *crh-1(ot1342)* animals of both sexes. (**E**) Quantification of PHB>AVA synaptic GRASP(*otIs839*) in wild-type and *crh-1(tz2)* males expressing PHB::GOA-1^gof^(*otEx7925* and *otEx8158*). (**F** and **G**) Quantification of PHB>AVA synaptic GRASP(*otIs839*) in *crh-1(tz2)* (F) and *tph-1(ot1274)* (G) males with overexpressing CRH-1 missense allele transgenes (*otEx8045* for CRH-1^WT^, *otEx8046* for CRH-1^S48E^, and *otEx8082* for CRH-1^S48A^) in the PHB neurons. WT, wild type. (**H**) Schematic diagram indicates juvenile food experience acts through serotonin–G protein–coupled receptor–GOA-1 to activate CRH-1 to secure LIN-29A expression upon sexual maturation to establish PHB>AVA sexually dimorphic connectivity. Statistics: [(A), (C), and (E) to (G)] One-way ANOVA and [(B), (D), and (I)] two-way ANOVA followed by Bonferroni multiple comparisons test. *P* value and *n* numbers are indicated on the graph. Scale bars, 10 μm. + indicates the mean value.

We have previously shown that juvenile starvation disrupts serotonin signaling through the metabotropic serotonin receptor SER-4 to control PHB>AVA en passant synapse number growth and elimination (*5*). To dissect the serotonin-triggered signaling cascade in the PHB neuron, we expressed specifically in the PHB neurons a gain-of-function version (gof) of the G-alpha protein, GOA-1, that we hypothesized to act downstream of the SER-4 G protein–coupled serotonin receptor (*17*). We found that the growth of PHB>AVA synapse number in adult males that were starved at the L1 stage (resulting in a loss of sexual dimorphism) was suppressed in such transgenic animals (Fig. 2A), indicating that GOA-1 signaling in male PHB neurons promotes the male-specific diminishment of PHB>AVA en passant synapses. This result is further substantiated by genetic removal of the 5-HT–synthesizing enzyme, TPH-1. In *tph-1* mutant males, sexually dimorphic PHB>AVA connectivity was disrupted, based on GRASP, CLA-1, and AVR-14 punctae (Fig. 2B and fig. S1, C and D), and these defects were rescued by PHB-specific GOA-1^gof^ expression (Fig. 2C).

Among the many downstream effector pathways of metabotropic 5-HT signaling is the activation of CREB via protein phosphorylation (*10*, *11*, *18*, *19*). We analyzed two independent *crh-1/CREB* loss of function alleles, *tz2* and *ot1342*, and found that the sexually dimorphic nature of the PHB>AVA connection was lost in these animals (Fig. 2D). PHB-specific GOA-1^gof^ failed to rescue ectopic PHB>AVA synaptic defects in *crh-1/CREB* mutants (Fig. 2E), confirming that CRH-1/CREB functions downstream of 5-HT–G protein–coupled receptor signaling to mediate adult male-specific PHB>AVA remodeling. We also made use of the fact that CREB proteins, including *C. elegans* CRH-1, are activated by upstream G protein signaling via a defined phosphorylation site, serine-48 (S48) in *C. elegans* (S133 in mammalian CREB) (*11*, *20*). A phosphorylation-deficient mutation (S48A) is predicted to inactivate the protein, while a phosphomimetic mutation (S48E) is predicted to make CRH-1/CREB independent of an upstream-activating input (in this case, loss of serotonin signaling). We found that restoration of wild type and CRH-1^S48E^ but not CRH-1^S48A^ in the PHB rescued PHB>AVA defects in the *crh-1* mutant males (Fig. 2F). Moreover, only restoration of phosphomimetic CRH-1^S48E^ but not wild type or CRH-1^S48A^ into the PHB neuron rescued the PHB>AVA synaptic defects of serotonin-deficient *tph-1* mutant males (Fig. 2G), consistent with CRH-1 acting downstream of serotonin (Fig. 2H).

### CRH-1/CREB controls feeding state-dependent male-specific LIN-29A expression

**Fig. 3.**
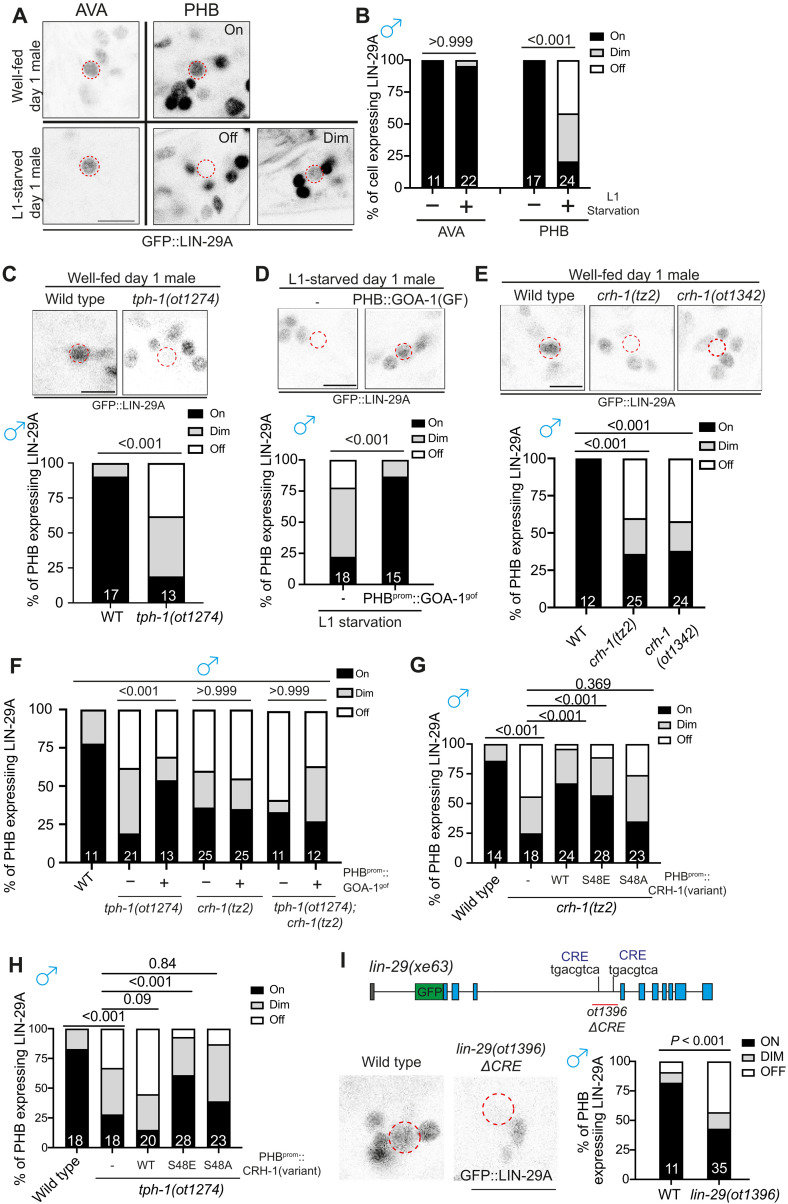
Male-specific LIN-29A expression in PHB is controlled by juvenile serotonin experience via CRH-1/CREB. (**A**) *lin-29(xe63[gfp::lin-29a])* expression of well-fed and L1-starved day 1 males in the AVA and PHB neurons. *lin-29(xe63[gfp::lin-29a])* expression is not affected in the AVA but is dim or lost in the PHB neurons when males undergo L1 starvation. (**B**) Quantification of the percentage of neurons expressing *lin-29(xe63[gfp::lin-29a])* in AVA or PHB under well-fed or L1 starvation conditions. (**C**) Representative images (top) and quantification (bottom) of PHB neurons expressing *lin-29(xe63[gfp::lin-29a])* in wild type and *tph-1(ot1274)*. (**D**) Representative images (top) and quantification (bottom) of PHB neurons expressing *lin-29(xe63[gfp::lin-29a])* in males that undergo L1 starvation with transgene overexpressing GOA-1^gof^ (*otEx8037*) in the PHB neurons. (**E**) Representative images (top) and quantification (bottom) of PHB neurons expressing *lin-29(xe63[gfp::lin-29a])* in wild type, *crh-1(tz2)*, and *crh-1(ot1342)*. (**F**) Quantification of PHB neurons expressing *lin-29(xe63[gfp::lin-29a])* in animals with or without PHB::GOA-1^gof^ transgene (*otEx8037*) overexpression in wild-type, *tph-1(ot1274)*, *crh-1(tz2)*, and *tph-1(1274); crh-1(tz2)* background. (**G**) Quantification of PHB neurons expressing *lin-29(xe63[gfp::lin-29a])* in *crh-1(tz2)* mutants with overexpressing CRH-1 missense allele transgenes (*otEx8053* for CRH-1^WT^, *otEx8157* for CRH-1^S48E^, and *otEx8113* for CRH-1^S48A^) in the PHB neurons. (**H**) Quantification of PHB neurons expressing *lin-29(xe63[gfp::lin-29a])* in *tph-1(ot1274)* mutants, overexpressing distinct types of *crh-1* transgenes (*otEx8053* for CRH-1^WT^, *otEx8157* for CRH-1^S48E^, and *otEx8113* for CRH-1^S48A^) in the PHB neurons. (**I**) Top: Schematic illustration of “CREB responsive element” (CRE) sites in the *lin-29a* locus. The *lin-29(ot1396)* allele is designed to delete the potential CRE sites at the intron 3 of the *lin-29a* locus. Bottom: Representative images (left) and quantification of (right) PHB neurons expressing GFP::LIN-29A in wild-type and *lin-29(ot1396)*. Statistics: Chi-square tests followed by Bonferroni multiple comparisons test. *P* value and *n* numbers are indicated on the graph. The red dashed circle indicates PHB. Scale bars, 5 μm.

We identified a functionally relevant transcriptional target of CRH-1/CREB by turning to an isoform of the Zn finger transcription factor LIN-29A, which we had previously shown to be expressed in several neuron classes, including PHB and AVA, only in males but not in hermaphrodites (*21*). We found that juvenile experience affects proper LIN-29A expression in sexually mature males. Starving animals at the L1 stage, transferring them back to food, and examining the expression of a CRISPR-Cas9–engineered reporter allele of *lin-29a* reveals an obvious decrease (reduction or complete elimination) of LIN-29A protein expression in 80% of analyzed animals (Fig. 3, A and B). We mimicked the starvation condition by supplementing well-fed animals with octopamine, an invertebrate catecholamine released under stress conditions, including starvation (*22*), and found that such treatment also reduced LIN-29A expression, hence recapitulating the effect of starvation (fig. S2A). Corroborating the previously reported critical window period at which starvation affects PHB>AVA synaptic remodeling (fig. S1E) (*5*), we observed that octopamine exposure at L1 but not the L3 stage results in reduced LIN-29A protein expression (fig. S2A). On the other hand, exogenously supplying 5-HT during L1 starvation rescued the LIN-29A expression deficiency (fig. S2A). This result is further confirmed by the demonstration that genetic removal of endogenous 5-HT by using animals that lack the 5-HT–synthesizing enzyme, TPH-1, diminishes LIN-29A expression in PHB (Fig. 3C). PHB-specific GOA^gof^ overexpression rescued the L1-starvation LIN-29A expression defects (Fig. 3D).

Male-specific LIN-29A expression in PHB was also diminished in two independent *crh-1/CREB* alleles (Fig. 3E). PHB-specific GOA^gof^ overexpression restored LIN-29A expression defects in *tph-1* mutants but not in *crh-1* or *tph-1; crh-1* double-mutant males, consistent with GOA-1 acting downstream of serotonin but upstream of CRH-1/CREB activation (Fig. 3F). Restoration of wild type and CRH-1^S48E^ but not CRH-1^S48A^ CRH-1 rescued LIN-29A expression defects in the *crh-1* mutant males (Fig. 3G). Moreover, only restoration of phosphomimetic CRH-1^S48E^ but not wild type or CRH-1^S48A^ rescued the reduction of LIN-29A expression in serotonin-deficient *tph-1* mutant males (Fig. 3H). The effect of CRH-1/CREB in LIN-29A expression is likely to be direct, because deletion of putative “CREB responsive elements” (CREs) in the third intron of the *lin-29a* gene phenocopied defective LIN-29A expression in *crh-1/CREB* mutant animals (Fig. 3I).

### LIN-29A expression is intersectionally controlled by sexual, spatial, and temporal specificity factors

**Fig. 4.**
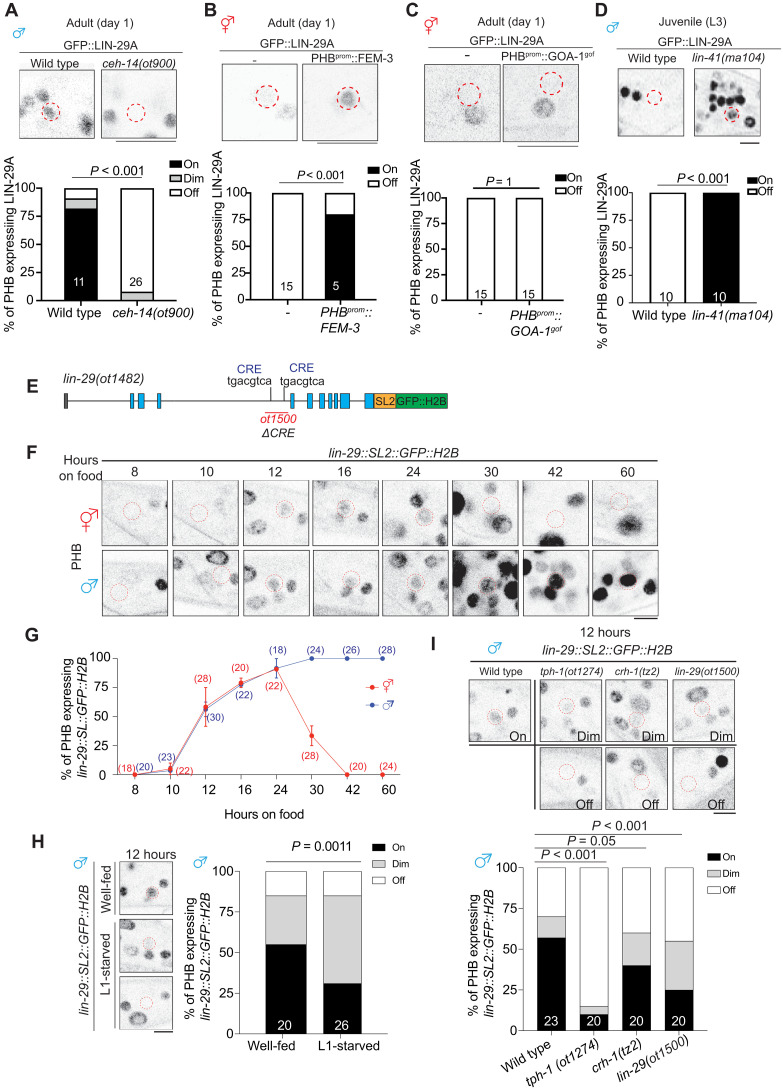
Early larval *lin-29a* transcription is regulated by serotonin>CREB signaling. (**A**) Representative images (top) and quantification (bottom) of PHB neurons expressing *lin-29(xe63[gfp::lin-29a])* in wild-type and *ceh-14(ot900)* males. (**B**) Representative images (top) and quantification (bottom) of PHB expressing *lin-29(xe63[gfp::lin-29a])* with transgene masculinizing PHB (PHB::FEM-3)(*otEX7916*) in hermaphrodites. (**C**) Representative images (top) and quantification (bottom) of PHB expressing *lin-29(xe63[gfp::lin-29a])* in hermaphrodites with transgene overexpressing GOA-1^gof^ (*otEx8037*) in the PHB. (**D**) Representative images (top) and quantification (bottom) of PHB expressing *lin-29(xe63[gfp::lin-29a])* in L3 wild-type and *lin-41(ma104)* males. (**E**) Schematic illustration of *lin-29(ot1482[lin-29::SL2::GFP::H2B])* and *lin-29(ot1500).* The *lin-29(ot1500)* allele is designed to delete the trunk of DNA elements, including the potential CRE site at the intron 3 of the *lin-29a* locus in *lin-29(ot1482*). (**F** and **G**) Longitudinal analysis of *lin-29(ot1482[lin-29::SL2::GFP::H2B])* expression in PHB neuron. Representative images (F) and quantification (G) of expression of PHB neuron expressing *lin-29(ot1482[lin-29::SL2::GFP::H2B])* in different time points after food exposure. (**H**) Representative images (left) and quantification (right) of PHB neurons expressing *lin-29(ot1482[lin-29::SL2::GFP::H2B])* in males that undergo L1 starvation after 12 hours of food exposure. (**I**) Representative images (top) and quantification (bottom) of PHB neurons expressing *lin-29(ot1482[lin-29::SL2::GFP::H2B])* in wild type, *tph-1(ot1274)*, *crh-1(tz2)*, and *lin-29(ot1500)* males after 12 hours of food exposure. Statistics: Chi-square tests followed by Bonferroni multiple comparisons test. *P* value and *n* numbers are indicated on the graph. The red dashed circle indicates PHB. Scale bars, 5 μm. + indicates the mean value.

The feeding state-dependent control of LIN-29A protein appearance in the male PHB neurons is the result of complex regulation of LIN-29A protein expression and raises the question of how a signal perceived at the L1 stage is translated into male-specific LIN-29A protein appearance at later larval stages. We investigated all axes of LIN-29 regulation, i.e., its cell-type/spatial specificity (PHB neuron), its sexual specificity (in males), its temporal specificity (protein appearance during sexual maturation), and feeding state dependence. We find that the terminal selector of PHB identity, the *ceh-14* LIM homeobox gene, the *C. elegans* homolog of vertebrate LHX3/4 (*23*, *24*), controls the cell-type specificity of induction of LIN-29A in PHB (Fig. 4A). The sexual specificity of LIN-29A induction in male PHB and not hermaphrodite PHB is, in turn, specified by the global master regulator of sexual identity, the Zn finger transcription factor TRA-1 (*25*, *26*), because removal of TRA-1 selectively in PHB results in LIN-29A depression in hermaphrodite PHB (Fig. 4B), in addition to neurons elsewhere in the nervous system (*21*). Two motifs located >10 kb away from the *lin-29a* transcriptional start site match experimentally determined TRA-1 binding sites (*27*). However, CRISPR-Cas9–mediated deletions of these sites do not result in ectopic *lin-29a* transcription in the day 1 hermaphrodite (fig. S2B), suggesting that TRA-1 operates either through nonconventional binding sites or indirectly via other factors. CREB activation cannot overcome TRA-1–dependent, sex-specific repression because the PHB::GOA-1^gof^ transgene is insufficient to induce ectopic LIN-29A protein expression (Fig. 4C) or ectopic synaptic elimination (fig. S6C) in hermaphrodites. The inability of PHB::GOA-1^gof^ to induce *lin-29a* expression in hermaphrodites in PHB also argues against the possibility that the recently reported TRA-1–mediated dampening of *goa-1* expression throughout the hermaphrodite nervous system (*28*) plays a causative role in mediating *lin-29a* repression in hermaphrodites. Taken together, we propose that activated CREB cooperates with the terminal selector of PHB identity, CEH-14/LHX3, to activate *lin-29a* transcription in PHB, and that this activation is antagonized in hermaphrodites by the master regulator of hermaphroditic sex, TRA-1.

Previous work has suggested that the temporal aspect of LIN-29A protein accumulation is controlled by the globally expressed heterochronic pathway, such that the translational inhibitor LIN-41, an RNA binding protein, represses *lin-29a* translation until the fourth larval stage (*21*, *29*, *30*). We observed precocious LIN-29A protein expression in the PHB neuron of *lin-41* mutant males (Fig. 4D). Therefore, we surmise that past feeding state is bookmarked by CREB on the level of *lin-29a* transcription at earlier larval stages, followed by translational inhibition of *lin-29a* transcripts by the heterochronic pathway. To investigate this issue further, we visualized *lin-29a* gene transcription (rather than LIN-29A protein production) by generating an SL2-based transcriptional *lin-29a* reporter through CRISPR-Cas9 genome engineering (Fig. 4E). We found that *lin-29a* transcription in PHB was induced in both sexes after animals were exposed to food for 12 hours (late L1 stage) and peaked at 24 hours (late L2 stage) (Fig. 4, F and G, and fig, S3). *lin-29a* transcription in another neuron that expresses LIN-29a protein in the adult, AVA, is not yet observed during these stages (figs. S2, C and D, and S3). In the hermaphrodite, *lin-29a* transcription began to be inhibited at the L3 stage, coinciding with the time when neuronal TRA-1 expression increases (*31*). The onset of *lin-29a* transcription after 12 hours of feeding in the late stage is reduced if animals have been starved before food exposure (Fig. 4H). Similarly, animals lacking either *tph-1*, *crh-1/CREB*, or the CRE site in the *lin-29a* transcriptional reporter allele show reduced *lin-29a* transcription in PHB (Fig. 4I). Such reduced *lin-29a* transcription was retained in day 1 adult males (fig. S2, E and F). Together, LIN-29A in the PHB acts as a hub by integrating not only temporal (heterochronic pathway), sexual (TRA-1), and spatial (i.e., cell-type specific) information (CEH-14/LHX3) but also an environmental axis that bookmarks past feeding status via CREB activation.

### LIN-29A is required to specify sexually dimorphic PHB>AVA synaptic connectivity

**Fig. 5.**
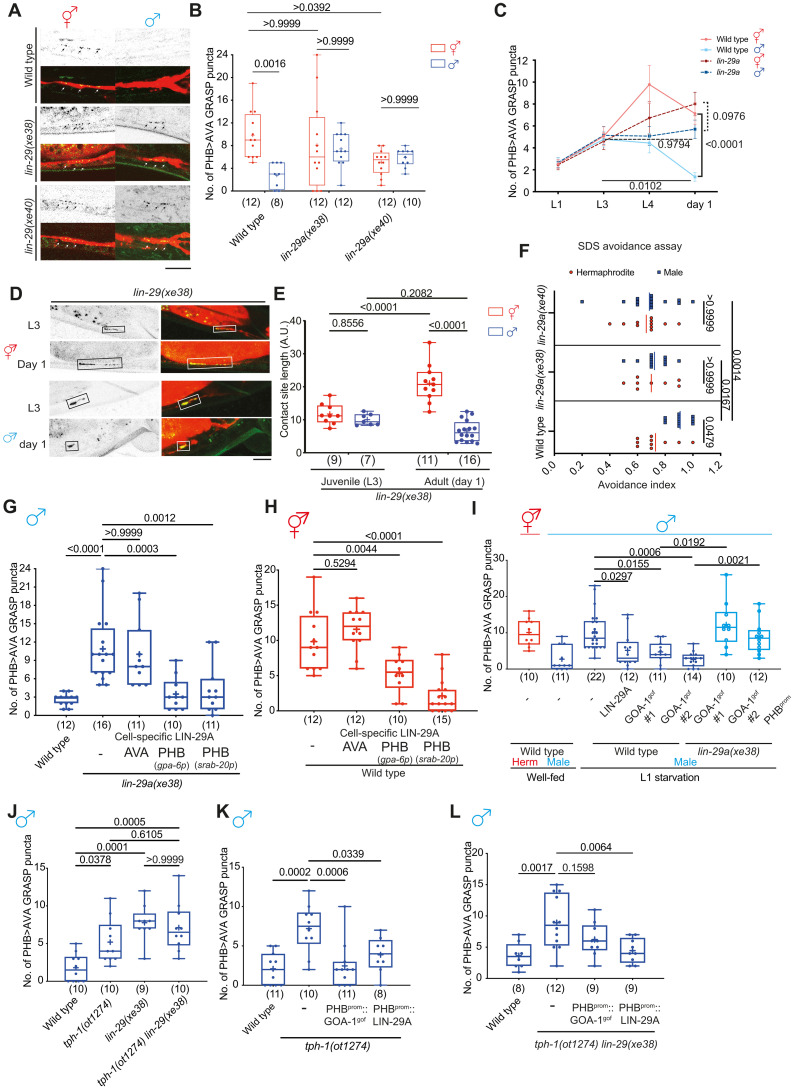
LIN-29A in PHB is required and sufficient to establish PHB>AVA sexual dimorphic connectivity upon sexual maturation. (**A** and **B**) Representative images (A) and quantification (B) of PHB>AVA synaptic GRASP(*otIs839*) in day 1 wild type, *lin-29(xe38)*, and *lin-29(xe40)* in both sexes. (**C**) Developmental analyses of PHB>AVA synaptic connectivity in wild-type and *lin-29a* mutants. *n* > 10 for each genotype and sex at any given time point. (**D**) Representative images of PHB>AVA neurite CD4-GRASP(*otEx8152*) in *lin-29a* mutants in both sexes. (**E**) Quantification of CD4-GRASP(*otEx8152*) *lin-29a* mutants in both sexes. (**F**) Quantification of SDS-avoidance assay in wild type, *lin-29(xe38)*, and *lin-29(xe40)*. (**G**) Quantification of PHB>AVA synaptic GRASP in *lin-29(xe38)* males with transgenes expressing LIN-29A cDNA in either AVA(*otEx7763*) or PHB (*otEx7790* for *gpa-6p* and *otEx7915* for *srab-20p*). (**H**) Quantification of PHB>AVA synaptic GRASP in wild-type hermaphrodite with transgenes expressing LIN-29A cDNA in either AVA(*otEx7763*) or PHB (*otEx7790* for *gpa-6p* and *otEx7915* for *srab-20p*). (**I**) Quantification of PHB>AVA synaptic GRASP(*otIs839*) in well-fed wild-type animals and L1-starved wild-type and *lin-29(xe38)* animals expressing PHB::LIN-29A (*otEx7915*) or PHB::GOA-1^gof^(*otEx7925* and *otEx8158*). (**J**) Epistasis analysis of *tph-1* and *lin-29a* for PHB>AVA synaptic GRASP(*otIs839*) in males. (**K** and **L**) Quantification of PHB>AVA synaptic GRASP in *tph-1(ot1274)* (K) and *tph-1(ot1274) lin-29(xe38)* (L) males with transgene overexpressing GOA-1^gof^ (*otEx7925)* and LIN-29A(*otEx7915*) in the PHB. Statistics: [(B), (E), (F), and (I)] Two-way ANOVA, (C) three-way ANOVA, and [(G), (H), and (J) to (L)] one-way ANOVA followed by Bonferroni multiple comparisons test. *P* value and *n* numbers are indicated on the graph. Scale bars, 10 μm. + indicates the mean value.

Having shown that early-life serotonin signaling is a critical inducer of LIN-29A expression, we next asked whether *lin-29a* mutant males phenocopied L1-starvation effects on sexually dimorphic synaptic connectivity. We found that the sexually dimorphic nature of PHB>AVA synapses was abolished by two independent *lin-29a* null alleles, *xe38* and *xe40.* These defects can be measured with a GRASP transgene, as well as presynaptic CLA-1 and postsynaptic AVR-14 markers (Fig. 5, A to C, and fig. S4). Sexually dimorphic connectivity of other LIN-29–expressing neurons is not affected (fig. S5). Consistent with the L1 starvation results, PHB/AVA neurite contact length was also not affected in *lin-29a* mutant animals (Fig. 5, D and E).

We assessed the behavioral consequences of loss of *lin-29a* by considering the physiological function of PHB>AVA en passant synapses, which lie in mediating an avoidance response to noxious chemicals such as SDS (*32*). This response is sexually dimorphic in day 1 adult animals such that hermaphrodites avoid SDS less than their male counterparts due to sex-specific PHB>AVA connectivity (*6*). We quantified the avoidance response in *lin-29a* mutant males and found that the response was feminized (Fig. 5F).

Because a *lin-29a* reporter allele is expressed in both presynaptic PHB and postsynaptic AVA (*21*), we addressed the cellular focus of action of LIN-29A through cell-specific rescue experiments and found that restoration of LIN-29A in only the PHB neurons but not the AVA neurons restored proper synaptic elimination in *lin-29a* mutant males (Fig. 5G). Moreover, overexpressing LIN-29A in the PHB neuron in wild-type hermaphrodites caused ectopic PHB>AVA synaptic loss (Fig. 5H). We corroborated that *lin-29a* functions in PHB downstream of early juvenile food experience by showing that the rescuing effect of GOA-1^gof^ in starved males genetically depends on *lin-29a* (Fig. 5I). Furthermore, genetic removal of *lin-29a* in serotonin-deficient *tph-1* mutant males did not further increase the synaptic defects compared to that of either *tph-1* or *lin-29a* single-mutant animals (Fig. 5J). Overexpression of either GOA-1^gof^ or LIN-29A in the PHB rescued the defects in serotonin-deficient *tph-1* males (Fig. 5K), while overexpression of LIN-29A but not GOA-1^gof^ rescued the defects in *tph-1 lin-29a* double mutants (Fig. 5L). Last, the effect of masculinization of PHB through PHB-specific TRA-1 removal on PHB>AVA connectivity (*6*) is suppressed by *lin-29a* removal (fig. S6A) but not the effect of AVA masculinization (fig. S6B). Together, our results suggest that LIN-29A in the PHB sensory neurons acts downstream of early juvenile food experience and is required and sufficient to promote synaptic elimination in males.

We investigated the structural requirements of LIN-29A function. We found that the DNA binding activity of LIN-29A was required for its function because LIN-29A with Zn finger domain deletion failed to rescue the *lin-29a* defects and was insufficient to induce ectopic synaptic elimination (fig. S7, A and B). We also found that an otherwise uncharacterized human ortholog of LIN-29A, ZNF362, is able to rescue the synaptic elimination defects in *lin-29a* mutants (fig. S7C). Moreover, human ZNF362 is also able to induce ectopic synaptic elimination in wild-type hermaphrodites, just as LIN-29A (fig. S7D).

### LIN-29A represses DMD-4 in PHB to promote sexually dimorphic PHB>AVA connectivity

**Fig. 6.**
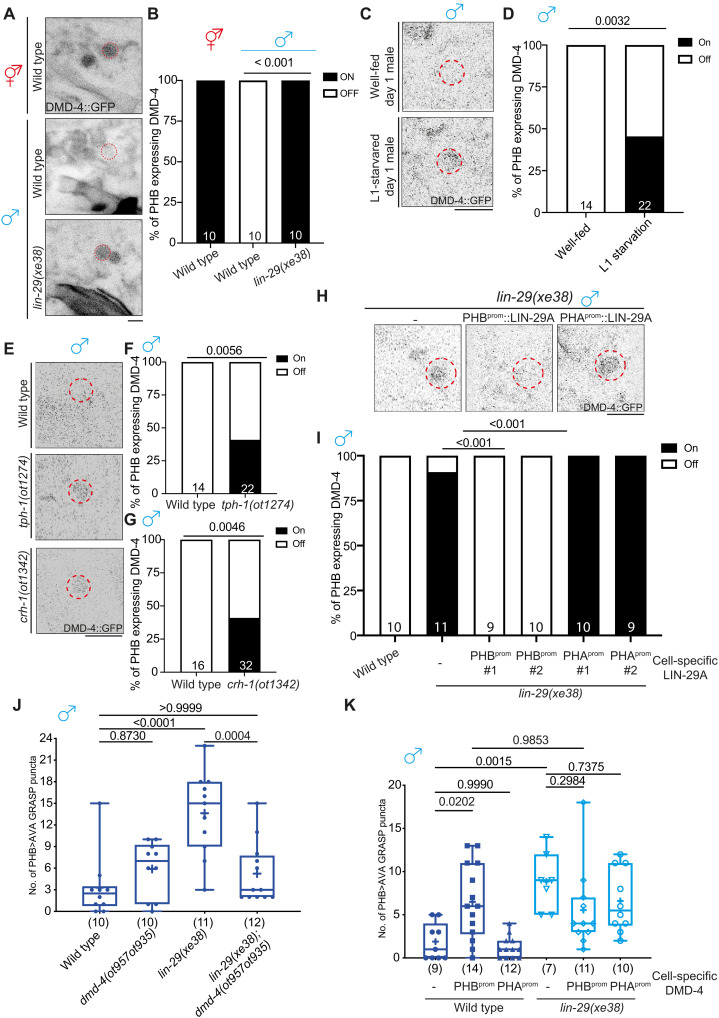
LIN-29A represses DMD-4 in PHB to control sexually dimorphic PHB>AVA connectivity. (**A** and **B**) Representative images (A) and quantification (B) of PHB neurons expressing *dmd-4(ot935)* in wild-type hermaphrodite and male and *lin-29(xe38)* male. (**C** and **D**) Representative images (C) and quantification (D) of PHB neurons expressing *dmd-4(ot935)* in well-fed and L1-starved day 1 males. (**E** to **G**) Representative images (E) and quantification [(F) and (G)] of PHB neurons expressing *dmd-4(ot935)* in wild-type, *tph-1(ot1274)*, and *crh-1(1342)* males. (**H** and **I**) Representative images (H) and quantification (I) of PHB neuron expressing *dmd-4(ot935)* in *lin-29(xe38)* with transgenes that express LIN-29A cDNA in either PHB *(otEx7961* and *otEx7964)* or PHA *(otEx8159* and *otEx8160)*. (**J**) Epistasis mutant analysis of *lin-29a* and *dmd-4* for PHB>AVA synaptic GRASP*(otIs839)* in males. (**K**) Quantification of PHB>AVA synaptic GRASP*(otIs839)* in wild-type and *lin-29(xe38)* males with transgene overexpression DMD-4 in either PHB (*otEx7984*) or PHA(*otEx7983*). Statistics: [(B), (D), (F), (G), and (I)] Two-proportion *Z* test and [(J) and (K)] one-way ANOVA followed by Bonferroni multiple comparisons test. *P* value and *n* numbers are indicated on the graph. The red dashed circle indicates PHB. Scale bars, 5 μm.

Several sex-specific animal features are controlled by phylogenetically conserved Doublesex/Mab-3–related transcription factors (*33*). We had previously shown that the DMD-4 protein, one of several *C. elegans* Doublesex homologs, most closely related to vertebrate DMRT3, is initially expressed in juvenile PHA and PHB neurons in both sexes but becomes selectively degraded in male PHA and PHB neurons upon sexual maturation (*12*). The mutually exclusive expression pattern of LIN-29A (males) and DMD-4 (hermaphrodites) in PHB led us to investigate whether *lin-29a* may control DMD-4 degradation in male PHB. We observed that DMD-4::GFP protein fails to be degraded in PHB in a *lin-29a* mutant background (Fig. 6, A and B). Because LIN-29A expression is feeding state dependent, we predicted that L1 starvation (which leads to loss of LIN-29A expression) might stabilize DMD-4 protein expression in PHB and found this to be the case (Fig. 6, C and D). Consistent with the implication of *tph-1* and *crh-1* in promoting LIN-29A expression, DMD-4 expression in PHB was also stabilized in well-fed day 1 *tph-1* and *crh-1/CREB* mutant males (Fig. 6, E to G). Cell-specific rescue experiments showed that *lin-29a* functions cell-autonomously in PHB to degrade DMD-4 in males (Fig. 6, H and I). We also find that PHB-specific expression of the human homolog of LIN-29A, ZNF362, rescues the effect of *lin-29a* on DMD-4 protein expression (fig. S7E).

As expected from DMD-4 expression in hermaphrodites (but not males), loss of *dmd-4* does not affect the lack of PHB>AVA synapse number growth in males (Fig. 6J). However, it is conceivable that it is the normal absence of *dmd-4* that accounts for diminished PHB>AVA synapse number in males and that, hence, the derepression of DMD-4 in *lin-29a* mutants is responsible for the ectopic PHB>AVA synapses in males. To test this notion, we generated *lin-29a; dmd-4* double-mutant animals and found that the synaptic defects found in *lin-29a* mutants were suppressed (Fig. 6J). Similarly, overexpressing DMD-4 in PHB neurons promoted the formation of en passant PHB>AVA synapses in males (Fig. 6K). Loss of *lin-29a* did not further enhance the ectopic synaptic defect in males overexpressing DMD-4 in PHB, consistent with the epistatic relationship of these genes. Together, *lin-29a* acts through the DMD-4 transcription factor to specify the sexually dimorphic nature of PHB>AVA en passant synapses.

### LIN-29A represses DMD-4 to inhibit *fmi-1* expression in adult male PHB

**Fig. 7.**
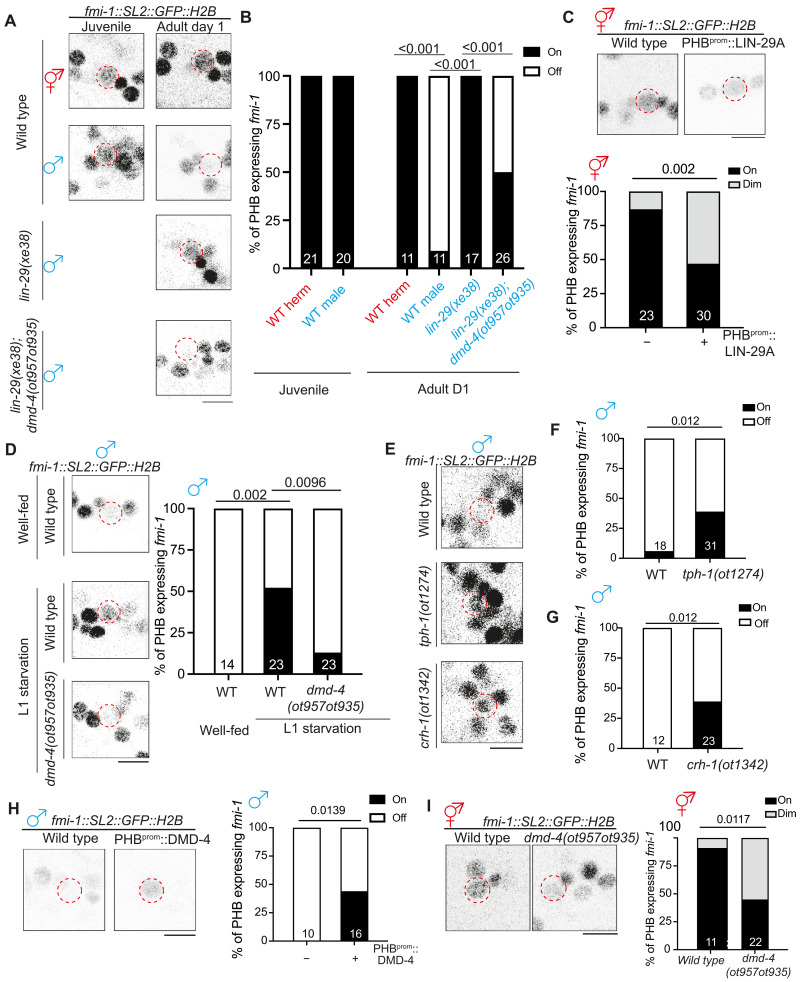
LIN-29A inhibits *fmi-1/Flamingo* expression via repressing DMD-4. (**A** and **B**) Representative images (A) and quantification (B) of *fmi-1(syb4563)* expression in the PHB in early L4 animals and day 1 animals with various genotypes. (**C**) Representative images (top) and quantification (bottom) of *fmi-1(syb4563)* expression in the PHB in wild-type hermaphrodites with transgene overexpressing LIN-29A cDNA(*otEx7961*) in the PHB neurons. (**D**) Representative images (left) and quantification (right) of *fmi-1(syb4563)* expression in the PHB in males that underwent L1 starvation. (**E** to **G**) Representative images (E) and quantification [(F) and (G)] of *fmi-1(syb4563)* expression in the PHB in wild-type, *tph-1(ot1274)*, and *crh-1(ot1342)* males. (**H**) Representative images (left) and quantification (right) of *fmi-1(syb4563)* expression in the PHB in wild-type males with transgene overexpressing DMD-4 cDNA (*otEx8083*) in the PHB neurons. (**I**) Representative images (left) and quantification (right) of *fmi-1(syb4563)* expression in the PHB in *dmd-4(ot957ot935)* hermaphrodites. Statistics: [(B) to (D) and (F) to (I)] Two-proportion *Z* test, followed by Bonferroni multiple comparisons test. *P* value and *n* numbers are indicated on the graph. The red dashed circle indicates PHB. Scale bars, 5 μm. + indicates the mean value.

We identified a functionally relevant effector gene for the CREB>LIN-29>DMD-4 regulatory cassette through a nervous system-wide expression pattern analysis of putative synaptogenic molecules, including all members of the cadherin gene family (*34*). We found that the unconventional cadherin protein FMI-1, the *C. elegans* homolog of the *Drosophila* Flamingo and vertebrate CELSR (cadherin EGF LAG seven-pass G-type receptors) proteins (*35*), showed a sexually dimorphic expression pattern in the PHB neurons of adult animals (Fig. 7, A and B). At juvenile stages, an SL2::GFP::H2B-based *fmi-1/CELSR* reporter allele, generated by CRISPR-Cas9 genome engineering, showed non-dimorphic expression in PHB neurons, but, upon sexual maturation, it was sex-specifically down-regulated in males. Other neurons in vicinity to PHB show no sex-dependent difference in *fmi-1/CELSR* expression (Fig. 7A). Sexually dimorphic *fmi-1/CELSR* expression is not only apparent on the transcriptional level (as measured with our SL2-based reporter allele) but also can be observed on the protein level. To visualize endogenous FMI-1/CELSR protein specifically in PHB, we engineered six copies of split GFP11(6xGFP11) at the C terminus of *fmi-1/CELSR* at the endogenous locus and overexpressed the other half of GFP with myristoylation peptide (myri-GPF1-10) in PHB (fig. S8, A and B). We found that reconstituted FMI-1::GFP intensity was significantly lower in day 1 males compared to their hermaphrodite counterparts, consistent with decreased *fmi-1/CELSR* transcription in males upon sexual maturation (fig. S8, C and D).

Male-specific down-regulation of *fmi-1/CELSR* gene and FMI-1/CELSR protein expression was lost in *lin-29a* mutants (Fig. 7, A and B, and fig. S8). Expression of either LIN-29A in PHB or its vertebrate homolog, ZNF362, rescued the ectopic expression of FMI-1/CELSR in male PHB (fig. S7F). Ectopic expression of LIN-29A in hermaphrodite PHB shows that LIN-29A is not only required but also sufficient to down-regulate *fmi-1/CELSR* expression (Fig. 7C). The feeding state dependence of LIN-29A expression predicts that the down-regulation of *fmi-1/CELSR* should also depend on the feeding state. We find that *fmi-1/CELSR* expression in male PHB is derepressed upon either L1 starvation or in well-fed serotonin-deficient *tph-1* or *crh-1/CREB* mutants (Fig. 7, D to F). The effect of *lin-29a* and feeding state on *fmi-1/CELSR* expression is mediated by the DMD-4 transcription factor because the ectopic expression of *fmi-1/CELSR* in male PHB in *lin-29a* mutants or after L1 starvation is suppressed by removal of *dmd-4* (Fig. 7D). These findings indicate that *dmd-4* normally acts to promote *fmi-1/CELSR* expression. Hermaphrodite-enriched PHB expression of *fmi-1/CELSR* is reduced in *dmd-4* mutants, and, conversely, overexpression of *dmd-4* in male PHB promotes *fmi-1/CELSR* expression (Fig. 7, H and I).

### FMI-1 separately controls neurite contact length and synaptogenesis 

**Fig. 8.**
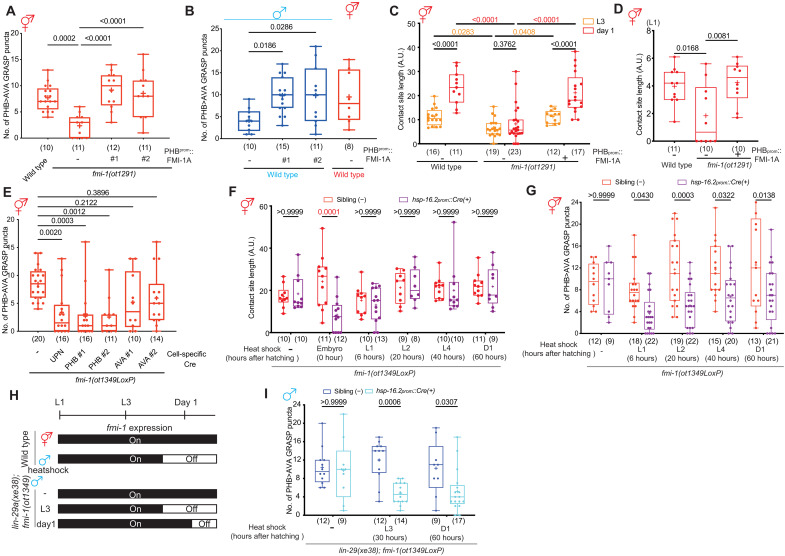
FMI-1 acts in PHB to promote the formation of en passant PHB>AVA synapses. (**A** and **B**) Quantification of PHB>AVA synaptic GRASP(*otIs839*) in *fmi-1(ot1291)* hermaphrodites (A) and wild-type males (B) with transgenes expressing FMI-1A cDNA (*otEx8032* and *otEx8033*) in the PHB neurons. (**C**) Quantification of CD4-GRASP(*otEx8152*) in L3 and day 1 wild-type, *fmi-1(ot1291)*, and fmi-1(ot1291); PHB::FMI-1A. (**D**) Quantification of CD4-GRASP(*otEx8152*) in L1 wild-type, *fmi-1(ot1291)*, and fmi-1(ot1291); PHB::FMI-1A. (**E**) Quantification of PHB>AVA synaptic GRASP(*otIs839*) in *fmi-1(ot1349)* hermaphrodite with transgenes expressing Cre pan-neuronally using the UPN driver (*50*) (*otEx8063*) or in PHB (*otEx8062* and *otEx8161*) or AVA (*otEx8064* and *otEx8162*). *fmi-1(ot1349)* is an *fmi-1* allele, which *fmi-1* locus is flanked with LoxP site and a GFP::H2B tagged at the C-terminal region. (**F**) Quantification of PHB>AVA synaptic GRASP(*otIs839*) in *fmi-1(ot1349)* hermaphrodites with transgenes expressing Cre in heat-shock promoter (*otEx8084*) and heat shock was performed by indicated time point. (**G**) Quantification of PHB>AVA synaptic GRASP(*otIs839*) in *fmi-1(ot1349)* hermaphrodites with transgenes expressing Cre in heat-shock promoter (*otEx8084*) and heat shock was performed by indicated time point. (**H**) Schematic illustration of *fmi-1* expression in the *lin-29(xe38); fmi-1(ot1349)* in heat-shock *fmi-1* removal experiment. (**I**) Quantification of PHB>AVA synaptic GRASP(*otIs839*) in *lin-29(xe38);fmi-1(ot1349)* males with transgenes expressing Cre in heat-shock promoter (*otEx8084*) and heat shock was performed by indicated time point. Statistics: [(A), (B), (D), and (E)] One-way ANOVA and [(C), (F), (G), and (I)] two-way ANOVA followed by Bonferroni multiple comparisons test. *P* value and *n* numbers are indicated on the graph. + indicates the mean value.

The regulation of *fmi-1/CELSR* by the serotonin>CREB>LIN-29A>DMD-4 axis, together with the documented role of vertebrate *fmi-1* orthologs in synaptogenesis (*36*), indicated that *fmi-1* may act as a synaptogenic molecule in the PHB>AVA context and that its serotonin/LIN-29A–mediated down-regulation suppresses PHB>AVA synapse number increases in males, hence generating synaptic sexual dimorphisms. We found that a *fmi-1/CELSR* null-mutant allele that we generated by CRISPR-Cas9 genome engineering results in decreased PHB>AVA GRASP puncta and presynaptic CLA-1 and postsynaptic AVR-14 puncta (Fig. 8A and fig. S9, A to F). Moreover, the PHB>AVA synaptic defects were rescued when FMI-1A was expressed in PHB. In males, FMI-1A ectopic expression in PHB also induced ectopic PHB>AVA synapses (Fig. 8B). However, we also noted that the extent of PHB and AVA neurite contact, measured with CD4-based GRASP, is significantly reduced as well (Fig. 8C), already at the first larval stage (Fig. 8D). The defect can be rescued by cell-specific reexpression in the PHB neurons (Fig. 8, C and D). This indicates that FMI-1 has, consistent with its role in other parts of the *C. elegans* nervous system (*35*, *37*), a role in neurite pathfinding and/or fasciculation of PHB during embryonic development, therefore not allowing us to conclude that synaptic defects in *fmi-1/CELSR* mutants are the result of sexually dimorphic synaptogenic defects during postembryonic development.

To address this issue, i.e., to separate embryonic from possible later roles of *fmi-1/CELSR*, we used a conditional gene removal strategy in which we inserted loxP sites into the reporter-tagged *fmi-1/CELSR* locus (fig. S8A). We first confirmed that continuous removal of *fmi-1* using pan-neuronal and PHB-specific but not AVA-specific Cre driver lines recapitulated synaptic defects in *fmi-1(ot1291)* null mutant (Fig. 8E). We removed *fmi-1/CELSR* in a temporally controlled removal manner using a heat-shock–inducible Cre driver line. Embryonic induction of Cre expression recapitulated the neurite contact length defects, while *fmi-1/CELSR* removal at any postembryonic stage had no effect on PHB/AVA contact length (Fig. 8F). In contrast, eliminating *fmi-1/CELSR* at postembryonic stages caused a decrease in PHB>AVA synapses in hermaphrodites compared to those of counterparts without transgene (Fig. 8G). Moreover, we found that removal of *fmi-1/CELSR* at the adult stage resulted in a reduction of PHB>AVA synapses, demonstrating that *fmi-1/CELSR* is not only required for synapse number increase during sexual maturation but also is continuously required to sustain synaptic connectivity in hermaphrodites.

Last, we asked whether ectopic en passant synapses in *lin-29a* mutant males result from derepressed synaptogenic *fmi-1/CELSR* function in PHB. To this end, we removed *fmi-1* postembryonically in *lin-29a* mutant to bypass its critical embryonic neurite placement function. We found that removal of *fmi-1/CELSR* in *lin-29a* mutant animals before sexual maturation (L3) and adulthood (day 1) significantly decreased the PHB>AVA synaptic puncta compared to the respective non-transgenic siblings (Fig. 8, H and I).

Together, these experiments demonstrate that FMI-1/CELSR has two separable functions: one during embryonic development in neurite outgrowth and placement and a synaptogenic one during postembryonic development. Because postembryonic expression of FMI-1/CELSR is sexually dimorphic, depending on the feeding state of the animal, we conclude that FMI-1/CELSR is the key synaptogenic effector gene of the CREB>LIN-29A>DMD-4 regulatory cascade. In hermaphrodites, this synaptogenic function is DMD-4–dependent and unimpeded by feeding state, while, in male animals, feeding state and LIN-29A–dependent suppression of FMI-1/CELSR expression results in the decrease in the number of sexually dimorphic en passant synapses.

## DISCUSSION

**Fig. 9.**
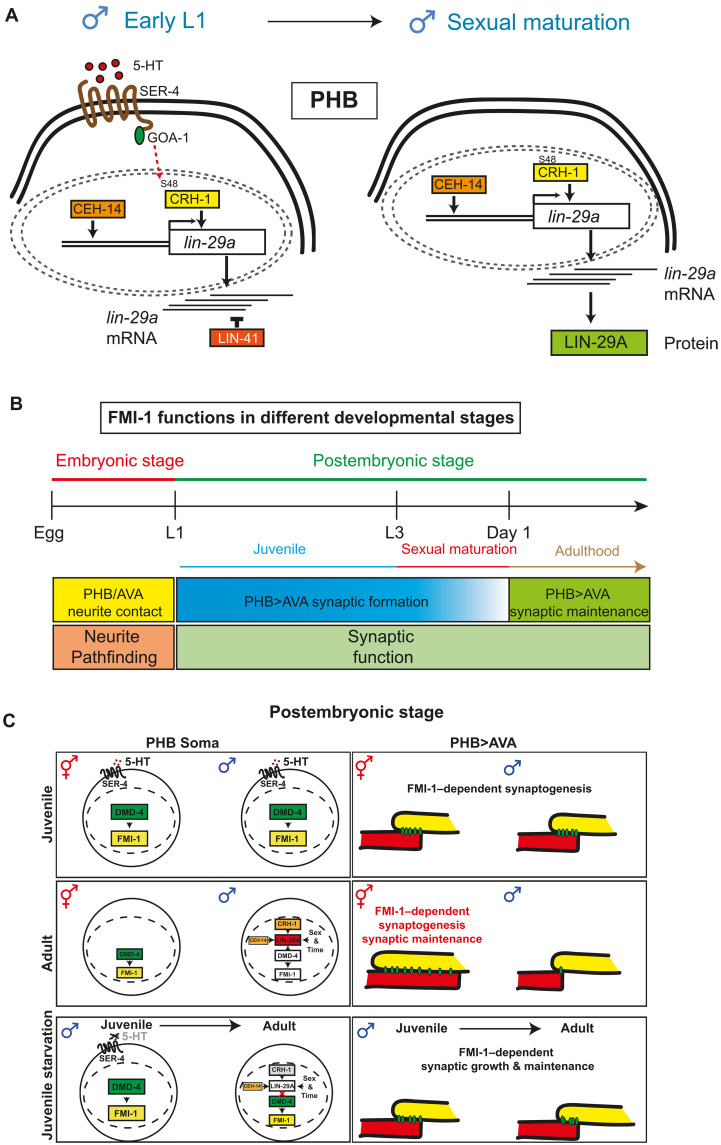
Schematic summary of results. (**A**) LIN-29A integrates four dimensions of information, including cell identity (CEH-14), sexual identity (TRA-1), temporal identity (LIN-41), and feeding state experience (serotonin–SER-4–CRH-1). (**B**) Schematic illustration of FMI-1 functions in PHB embryonic and postembryonic developmental stages and adulthood. (**C**) Schematic illustration of the regulation of LIN-29A>DMD-4>*fmi-1* in the nucleus and the corresponding regulation of PHB>AVA neurite adjacency and synaptogenesis.

Early-life experience affects later brain development or neurological disorders in a sex-specific manner, but the cellular and molecular mechanisms that lead to sex-specific vulnerability remain largely unknown. Here, we uncover the molecular basis for how the juvenile feeding-state experience affects the generation of sex specificity of synaptic connectivity between a nociceptive sensory neuron and a command interneuron target (summarized in Fig. 9).

A key regulatory bottleneck in this process is an evolutionarily conserved Zn finger transcription factor, LIN-29A, whose expression integrates four dimensions of specificity (Fig. 9). Transcription of the *lin-29a* locus is directed to a subset of neuronal cell types (including PHB) via terminal selectors, such as CEH-14/LHX3 (shown here). The male specificity of *lin-29a* transcription is imposed by the global sex identity regulator TRA-1, which antagonizes *lin-29a* transcription in hermaphrodites, while the temporal specificity of LIN-29A protein accumulation during sexual maturation is controlled by translational repression of *lin-29a* transcripts through the global heterochronic regulator *lin-41*. This repression is relieved upon sexual maturation by miRNA-mediated down-regulation of *lin-41.* The fourth dimension of *lin-29a* regulation is conferred by the feeding state of the animal. A serotonin- and CREB-dependent input is required specifically in the L1 stage to enable terminal selector-dependent induction of *lin-29a* transcription that is then translated into protein expression during sexual maturation via the relief of translational repression. Once the initial *lin-29a* transcription activation has been bookmarked, the locus becomes independent of a requirement for a feeding input (as evidenced by post-L1 starvation having no effect on LIN-29A expression). We speculate that CRH-1/CREB-dependent bookmarking may involve changes in the chromatin status of the *lin-29a* locus to enable PHB-specific expression of the locus throughout development. Together, our studies tie an early transcriptional event, mediated by stimulus-dependent transcription factor, CREB, to the sustained expression of a locus, *lin-29a*, that, later in life, relays this input into the modulation of synaptic connectivity and, hence, information flow in the nervous system (Fig. 9). Both the regulation of LIN-29A and its function appear to be highly cell-type specific, a likely reflection of (i) the presumptive complexity of the cis-regulatory elements that can activate LIN-29A via distinct factors in different cellular contexts and (ii) the presumptive need for LIN-29A to cooperate with cell-specific cofactors to control specific effector genes.

Our study not only provides critical insights into the mechanistic basis of sculpting the sexually dimorphic nature of synaptic connectivity but also reveals two distinct components of the establishment of sexually dimorphic connectivity: a sexually dimorphic increase in the adjacency of two neurites and a sexually dimorphic increase in the number of en passant synaptic connections. These two processes can be mechanistically uncoupled by *fmi-1/CELSR*, which does not affect the postembryonic increase in neurite adjacency but only affects the increase in en passant synapse number and, postdevelopmentally, maintains this synaptic connectivity. Considering the early function of FMI-1/CELSR in controlling initial PHB/AVA neurite fasciculation during embryonic development, it is intriguing to note the lack of a role of FMI-1/CELSR in controlling the sex-specific adjacency increase of the PHB and AVA neurites during postembryonic sexual maturation. This observation suggests that neurite contacts of the same two neurons can be regulated by distinct means during distinct stages of development. Moreover, the distinct functions of FMI-1/CELSR in embryonic fasciculation and postembryonic synaptogenesis and maintenance are a likely reflection of context-dependent association of FMI-1/CELSR proteins with distinct interaction partners.

In vertebrates, the CELSR orthologs of FMI-1 have been implicated in axon outgrowth as well as synapse formation (*36*, *38*–*42*), but a function in maintaining synaptic structure had not been described before. Our work uniquely places FMI-1/CELSR function, as well as its apparent dependence on past experience, in the context of sexually dimorphic synaptic connectivity. Vertebrate brains are thought to display sexually dimorphic features on multiple levels (*43*–*47*), although the cellular complexity of vertebrate brains has hampered the definition of such dimorphisms on a single neuron/single synapse level. We hope that our work will motivate a careful analysis of sexually dimorphic CELSR expression in vertebrate brains, which may provide a critical entry point to not only identify vertebrate sexual dimorphisms but also understand their genetic specification.

## MATERIALS AND METHODS

### *C. elegans* strains and handling

Worms were grown at 20°C on nematode growth medium (NGM) plates seeded with *Escherichia coli* (OP50) bacteria as a food source unless otherwise mentioned. Worms were maintained according to standard protocol. *him-8(e1489)* and *him-5(e1490)* were used as wild type in this study to generate sufficient males. Table S1 lists a complete list of strains and transgenes generated and used in this study.

### CRISPR-Cas9–based genome engineering

To generate *tph-1(ot1274)*, two guide CRIPSR RNA (crRNAs) (5′-catcggatatctaaaagagg-3′ and 5′-acctctcttcatctcaatat-3′) and single-stranded oligodeoxynucleotides (ssODN) (5′-gtgccgaattccagaagcaccacgccatcggatatctaaaagaggccaacacaaagacacgttttcctgcagaagaggaa-3′) were used to remove the whole tph-1 locus. To generate *crh-1(ot1343)*, two guide crRNAs (5′-taaggagattagttttccaa-3′ and 5′-ttggagatttcttgttgagg-3′) and ssODN (5′-gtgtttgtttttcaaagaatagcttatatatatgatgaaatctcgtttttatttttatttcctaattttt-3′) were used to remove the whole *crh-1* locus. To generate lin-29(ot1396), two guide crRNAs (5′-atttgaacccaatattgaat-3′ and 5′-gagttcattttgatttcacg-3′) and ssODN (5′-agtggtcaaagaaatttgagagaaaaagtgcggagcgtgaaatcaaaatgaactcggctatatttcggcc-3′) were used to remove the potential CRE site in the *lin-29a* locus. To generate *fmi-1(ot1090)* and *fmi-1(ot1291)*, two guide crRNAs (5′-ttgaatgtgaatgtcagtgg-3′ and 5′-TGATGCGTATTACACATATA-3′) were used to remove the whole *fmi-1* locus. To generate fmi-1(ot1349), crRNA (5′-aagaactgaccagctgccaa-3′) and ssODN (5′- ctgatacaaccctttgctcttttcacctcatatgtATAACTTCGTATAGCATACATTATACGAAGTTATcccgggttggcagctggtcagttcttcttccaaagagacgc-3′) were used to insert the first LoxP site at 5′ untranslated region (5′UTR) region, and second crRNA (5′-atcggaacaatgaacaagta-3′) and homemade LoxP::GFP::H2B ssODN via polymerase chain reaction and exonuclease were used to insert the second LoxP site. To generate *fmi-1(ot1429)*, crRNA (5′-TACCACATCTACATTCAACA-3′) and codon-optimized 6XGFP_11_ ssODN were used to remove the original stop codon and insert GFP_11_ at the C terminus of fmi-1 gene locus. To generate, lin-29(ot1482), crRNA (5′-ttatcggaatatgtgagttc-3′) and homemade SL2::GFP::H2B ssOND were used.

### Molecular cloning

To generate pCPL1(*srab-20p::goa-1^gof^::SL2::TagRFP*) and pCPL4(*srab-20p::goa-1^gof^::SL2::3XNLS::GFP*), upstream ~1.2-kb promoter from *srab-20*, which was amplified from (*srab-20p::GFP)*, the synthetic *goa-1^gof^* DNA fragment, and the SL2-backbone amplified from *pEAB42 (srg-13p::ser-4::SL2::tagRFP)* were ligated. To generate pCPL2(*gpa-6p::lin-29a::SL2::3XNLS::GFP*) and pCPL3(*srab-20p::lin-29a::SL2::3XNLS::GFP*), *srab-20p* and *gpa-6p* amplified from pCPL4 and pEAB3 (2.6 kb of the *gpa-6* promoter fused to GFP), respectively, and lin-29a cDNA (1.4 kb) and the SL2-base backbone amplified from pCPL4 were ligated. To generate pCPL5(*srab-20p::crh-1^WT^::SL2::3XNLS::GFP*), pCPL6(*srab-20p::crh-1^S48E^::SL2::3XNLS::GFP*), and pCPL7(*srab-20p::crh-1^S48A^::SL2::3XNLS::GFP*), crh-1 variants subcloned from gcy-8*p::crh-1^WT^*, gcy-8*p::crh-1^S48E^*, and gcy-8*p::crh-1^S48A^* (gifts from C.-L. Pan) and backbone amplified from pCPL4 were ligated. To generate pCPL8(*srab-20p::crh-1^WT^::SL2::TagRFP*), pCPL9(*srab-20p::crh-1^S48E^::SL2::TagRFP*), and pCPL10(*srab-20p::crh-1^S48A^::SL2::TagRFP*), *crh-1* variants were subcloned into pCPL1 backbone. To generate pCPL11(*srab-20p::lin-29a::SL2::TagRFP*) and pCPL12(*srg-13p::lin-29a::SL2::TagRFP*), lin-29a was subcloned into the backbone amplified from pCPL1 and pEAB42(*srg-13p::ser-4::SL2::TagRFP*), respectively. To generate pCPL13(*srab-20p::dmd-4::SL2::TagRFP*), *dmd-4* cDNA (0.8 kb) was amplified and subcloned into pCPL1. To generate pCPL14(*srab-20p::fmi-1a::SL2::3XNLS::GFP*), *fmi-1a* cDNA was amplified from *ser2prom3::fmi-1a* (gift from C.-L. Pan) and subcloned into pCPL4. To generate pCPL15(*UPN::3xNLS::Cre*), pCPL16(*srab-20p::3xNLS::Cre*), pCPL17(*flp-18p::3XNLS::Cre*), and pCPL23(*hsp16.2::3xNLS::Cre*), UPN, *srab-20p*, *flp-18p* (~1.4 kb), and *hsp16.2p* (~0.4 kb) were subcloned into pCC301(*rab-3p1::3xNLS::Cre*). To generate pCPL18[*srab-20p::gfp::cla-1(s)*], *srab-20p* were digested from pCPL1 by Sph I and Xma I restriction enzymes and ligated into the backbone of pMM13[*cat-4p::gfp::cla-1(s)*]. To generate pCPL19(*flp-18p::avr-14::TagRFP*), *avr-14* was amplified from *avr-14* plasmid (gift from M. Oren) and ligated into pCC45(*flp-18p::TagRFP*). To generate pCPL20(*srab-20p::znf362::SL2::3XNLS::GFP*) and pCPL21(*srab-20p::znf362::SL2::TagRFP*), synthetic codon-optimized human znf362 cDNA was subcloned into pCPL4 and pCPL1, respectively. To generate pCPL24(*srab-20p::myriGFP_1-10_::SL2::TagRFP*), split GFP_1-10_ was cloned with myristoylation sequences and ligated with pCPL1 backbone. To generate pCPL25(*srab-20p::CD4::GFP_1-10_*) and pCPL26(*flp-18p::CD4::GFP_11_*), *srab-20p* and *flp-18p* were digested from pCPL16 and pCPL17, respectively, and ligated to the backbone from *ser2prom3p::CD4:: GFP_1-10_* and *unc-17p::CD4:: GFP_11_* (gifts C.-L. Pan), respectively.

### L1 starvation assay

Animals with indicated genotype were synchronized by hypochlorite treatment of gravid adults followed by 12 hours in M9 at 20°C for embryos to hatch. Synchronized L1 animals were released onto the unseeded NGM plates for another 24 hours. L1-starved animals were then washed and transferred to seeded NGM plates.

### Heat-shock assay

OH18506, *him-5(e1490) fmi-1(ot1349); otIs839; otEx8084[hsp-16.2p::3xNLS::Cre::p10UTR]*; OH19023, *him-5(e1490) fmi-1(ot1349)/V;otEx8084[hsp-16.2p::3xNLS::Cre::p10UTR]*,*otEx8152[srab-20p::TagRFP, srab-20p::CD4::GFP1-10,flp-18p::TagRFP, flp-18::CD4::GFP11]*; and OH19231, *lin-29(xe38);fmi-1(ot1349) him-5(e1490); otIs839; otEx8084[hsp-16.2p::3xNLS::Cre]* animals were synchronized by hypochlorite treatment of gravid adults followed by 12 hours in M9 at 20°C for embryos to hatch. Synchronized L1 animals were released onto the seeded NGM plates, and heat shock was performed 6, 20, 40, and 60 hours after releasing onto the seeded plates (indicated as hours after hatching the figures). Animals were heat shocked at 34°C for 20 min, followed by 20 min of resting at 20°C three times to induce sufficient heat-shock response. PHB>AVA GRASP puncta and PHB/AVA adjacency were analyzed at day 1 stage for groups that received heat shock at 6, 20, or 40 hours after hatching. For groups that received heat shock at 60 hours after hatching, analyses of PHB>AVA GRASP puncta and PHB/AVA adjacency were conducted 24 hours after heat shock.

### SDS avoidance behavior

The SDS avoidance assay was based on procedures as described. To deliver the testing droplets, we pulled 10-μl glass capillary pipette (VWR International) by hand on the flame to reduce the diameter of the tip and mounted the capillary pipette on a rubber tubing and operated by mouth. We delivered a small drop of solution containing either the repellent (0.1% SDS in M13 buffer) or buffer [M13 buffer: 30 mM tris-HCl (pH 7.0), 100 mM NaCl, and 10 mM KCl] to near the tail of an animal while it moves forward. Once in contact with the tail, the drop surrounded the animal by capillary action and reached the anterior head region. Assayed worms were transferred individually to fresh and unseeded NGM plates. Each assay started by testing the animals with drops of M13 buffer alone. The response to each drop was scored as reversing or not reversing. The avoidance index is the number of reversal responses divided by the total number of trials. An interstimulus interval of at least 2 min was used between successive drops of the same animal.

### Microscopy

Worms were anesthetized in 100 mM sodium azide on the 5% agarose on glass slides. All images were acquired using a Zeiss confocal microscope (LSM 880 or LSM980). For synaptic GRASP and gene expression experiments, animals were imaged using 63× objective and with a fixed imaging setting. For CLA-1 and AVR-14 puncta experiments, animals were imaged using 40× objective and with a fixed imaging setting. For PHB-AVA adjacency CD4-GRASP experiments, animals were 40× objective and with a fixed imaging setting.

### Quantification and statistical analysis

#### Quantification of synaptic GRASP puncta

For all the synaptic GRASP, the images were acquired using 63× objective and with a fixed imaging setting either with LSM880 or LSM980. The raw images were unbiasedly analyzed with PysQi (*48*), the automatic puncta quantification software.

#### Quantification of synaptic iBLINC puncta

For iBLINC experiments, animals were imaged using a 63× objective with a fixed imaging setting with LSM880, and puncta were quantified by scanning the original full *Z*-stack for distinct dots in the area where the processes of the two neurons overlap.

#### Quantification of GFP::LIN-29A, DMD-4::GFP, and lin-29a and fmi-1 expression

For expression of translational and transcriptional *lin-29a* reporter constructs, images of *lin-29(xe63[gfp::lin-29a])* and *lin-29(ot1482[lin-29::SL2::GFP::H2B])*, animals with different mutant or transgene overexpression backgrounds were acquired using 63× objective with fixed imaging settings with either LSM880 or LSM980. The expression level is categorized into three tiers: on, dim, and off. Cells with GFP fluorescent intensity lower than 50% of the normal “on” cells are identified as “dim.”

For DMD-4::GFP quantification, images of *dmd-4(ot935)* animals with different mutant or transgene overexpression backgrounds were acquired using 63× objective with fixed imaging settings with either LSM880 or LSM980.

For *fmi-1* gene expression quantification, images of *fmi-1(syb4563)* animals with different mutant or transgene overexpression backgrounds were acquired using 63× objective with fixed imaging settings with either LSM880 or LSM980. Cells with GFP fluorescent intensity lower than 50% of the normal on cells are identified as dim.

#### Quantification of PHB and AVA contact CD4 GRASP

The contact site length between PHB and AVA processes in the CD4 reporter was quantified in Fiji ImageJ (*49*). Briefly, the entire *Z*-stack was scanned while tracing over the GFP^+^ region (where the PHB and AVA processes overlap) with a segmented line and then measuring the overall line length. In cases where the contact and resulting GFP signal was discontinuous, multiple lines were drawn, measured independently, and summed to yield the overall contact site length. For visualization purposes, figures contain a representative subset of the *Z*-stack reconstructed as maximum intensity projection using Zeiss Zen software to display the maximal PHB-AVA contact site.

#### Quantification of CLA-1 and AVR-14 puncta

GFP::CLA-1 and AVR-14::TagRFP puncta in the PHB and AVA, respectively, were quantified manually in Fiji by scanning the entire *Z*-stack and only scoring puncta co-localizing with cytoplasmic AVAp::RFP and cytoplasmic PHBp::GFP, respectively.

#### Quantification of the juxtaposition of the CLA-1 and AVR-14 puncta

For scoring the juxtaposition of the PHB GFP::CLA-1 and AVA AVR-14::RFP puncta, each *Z*-stack was first scanned in the region of interest to quantify all GFP::CLA-1 puncta. Next, AVR-14::TagRFP puncta directly adjacent to with CLA-1 puncta were scored. The juxtaposition index is calculated as follows: AVR-14::TagRFP juxtaposed with (CLA-1::GFP/Total GFP::CLA-1)*100%.
