# Supplementary material for: Experience-dependent, sexually dimorphic synaptic connectivity defined by sex-specific cadherin expression
Source: Sci Adv. 2024 Nov 13;10(46):eadq9183. doi: 10.1126/sciadv.adq9183 (PMC11559607; doi:10.1126/sciadv.adq9183)
Supplement: Supplementary file 1 — Figs. S1 to S9 Table S1 [file sciadv.adq9183_sm.pdf]

Science Advances
AAAS

Supplementary Materials for

Experience-dependent, sexually dimorphic synaptic connectivity defined by sex-specific cadherin expression

Chien-Po Liao *et al.*

Corresponding author: Chien-Po Liao, cl4102@columbia.edu; Oliver Hobert, or38@columbia.edu

Sci. Adv. **10**, eadq9183 (2024)
DOI: 10.1126/sciadv.adq9183

This PDF file includes:

Figs. S1 to S9
Table S1

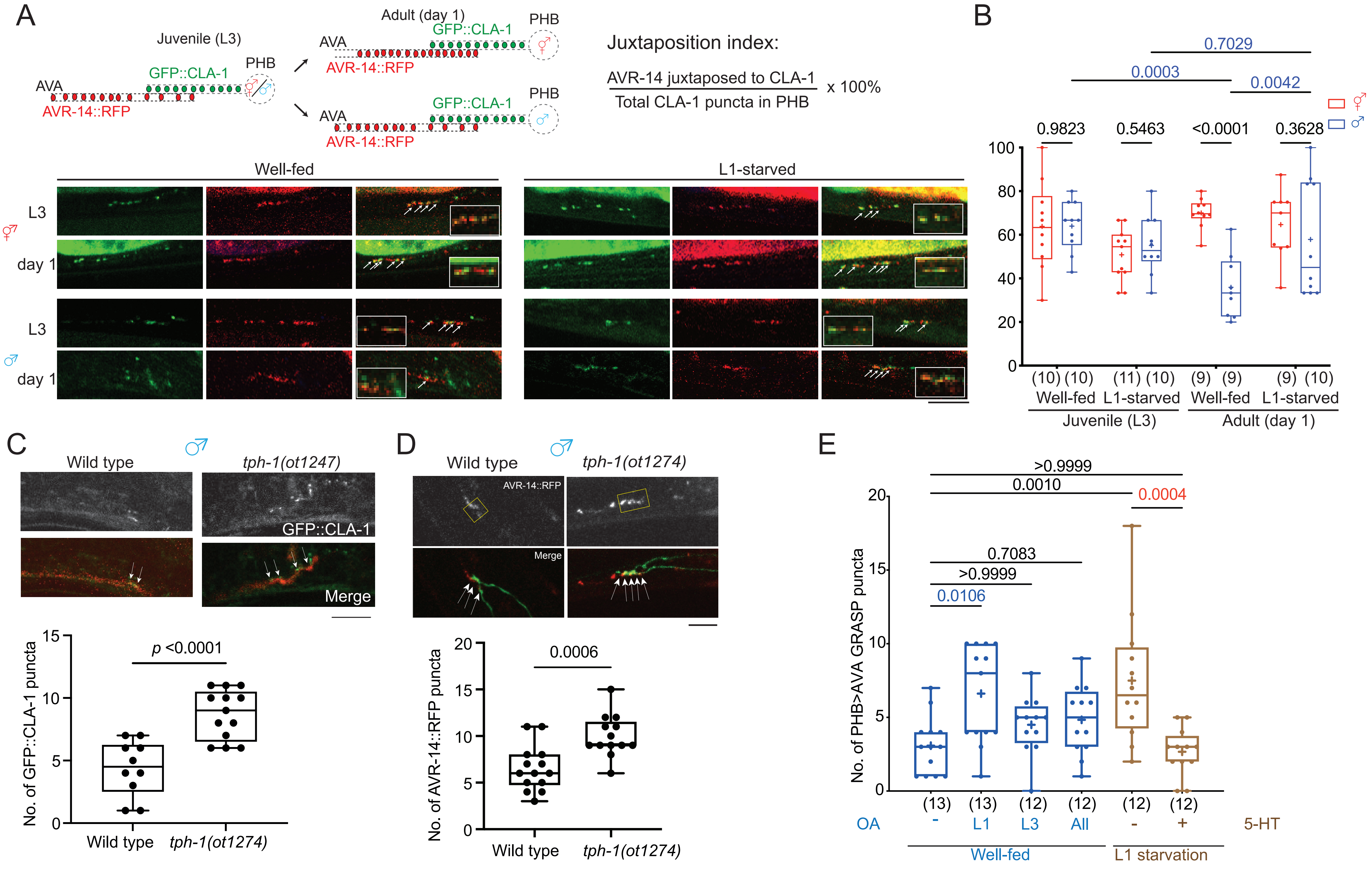
A
Juvenile (L3)
Adult (day 1)
AVA
PHB
GFP::CLA-1
AVR-14::RFP
Juxtaposition index:
AVR-14 juxtaposed to CLA-1
Total CLA-1 puncta in PHB
x 100%
Well-fed
L1-starved
L3
day 1
B
0.7029
0.0003
0.0042
0.9823
0.5463
<0.0001
0.3628
(10) (10)
(11) (10)
(9) (9)
(9) (10)
Well-fed
L1-starved
Juvenile (L3)
Adult (day 1)
C
Wild type
tph-1(ot1247)
GFP::CLA-1
Merge
No. of GFP::CLA-1 puncta
p <0.0001
Wild type
tph-1(ot1274)
D
Wild type
tph-1(ot1274)
AVR-14::RFP
Merge
No. of AVR-14::RFP puncta
0.0006
E
>0.9999
0.0010
0.0004
0.7083
>0.9999
0.0106
No. of PHB>AVA GRASP puncta
(13) (13) (12) (12) (12) (12)
OA
- L1 L3 All - +
5-HT
Well-fed
L1 starvation

Fig S1

Figure S1. Serotonin signaling affects PHB>AVA synaptic connectivity. Related to Figure 1 and 2.

(A) (top) Schematic illustration of the juxtaposition of GFP::CLA-1 in the PHB neuron and AVR-14::TagRFP in the AVA neuron in L3 and day 1 well-fed and L1-starved animals in both sexes. The juxtaposition index is calculated by the ratio of GFP::CLA-1 that juxtaposed to AVR-14::TagRFP to the total GFP::CLA-1 in the PHB, which normalizes the expression variability of the extrachromosomal array and reflects the proportion of the synapses that PHB forms onto AVA**.** (bottom)Representative images of the juxtaposition of PHB-localized GFP::CLA-1 and AVA-localized AVR-14::TagRFP (*otEx8163*) in L3 and day 1 well-fed and L1-starved animals in both sexes.

(B) Quantification of juxtaposition index in L3 and day 1 well-fed and L1-starved animals in both sexes.

(C) Representative images (top) and quantification (bottom) of AVA-juxtaposed GFP::CLA-1 in PHB (*otIs883;otEx8040*) in day 1 wild-type and *tph-1(ot1274)*.

(D) Representative images (top) and quantification (bottom) of PHB-juxtaposed AVR-14::TagRFP (*otIs902*) in day 1 wild-type and *tph-1(ot1274)*.

(E) Quantification of PHB>AVA synaptic GRASP (*otIs839*) in wild-type well-fed males supplemented with octopamine (OA) or L1-starved males supplemented with serotonin(5-HT) during the starvation period.

Statistics: (B) Two-way ANOVA followed by Bonferroni multiple comparisons test and (C,D) Student *t*-test. *p*-value and N numbers are indicated on the graph. Scale bar = 10 µm. + indicates the mean value.

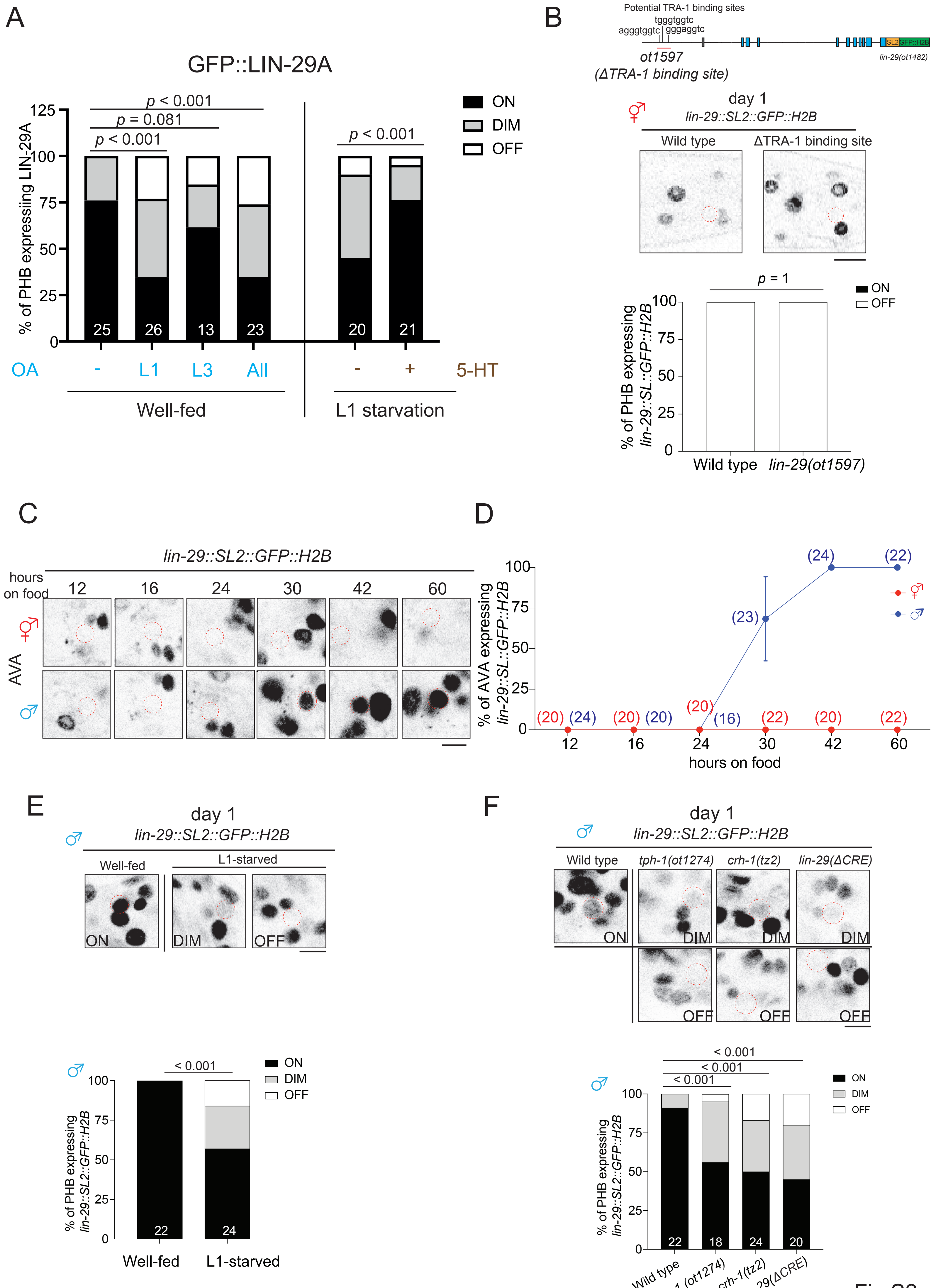

A
GFP::LIN-29A
% of PHB expressing LIN-29A
p < 0.001
p = 0.081
p < 0.001
p < 0.001
ON
DIM
OFF
25
26
13
23
20
21
OA
-
L1
L3
All
-
+
5-HT
Well-fed
L1 starvation
B
Potential TRA-1 binding sites
tgggtggtc
agggtggtc
gggaggtc
ot1597
(ΔTRA-1 binding site)
lin-29(ot1482)
day 1
lin-29::SL2::GFP::H2B
Wild type
ΔTRA-1 binding site
p = 1
ON
OFF
% of PHB expressing lin-29::SL::GFP::H2B
Wild type
lin-29(ot1597)
C
lin-29::SL2::GFP::H2B
hours on food
12
16
24
30
42
60
AVA
D
% of AVA expressing lin-29::SL::GFP::H2B
hours on food
(20) (24) (20) (20) (20) (16) (22) (20) (22) (23) (24) (22)
E
day 1
lin-29::SL2::GFP::H2B
Well-fed
L1-starved
ON
DIM
OFF
< 0.001
% of PHB expressing lin-29::SL2::GFP::H2B
22
24
Well-fed
L1-starved
F
day 1
lin-29::SL2::GFP::H2B
Wild type
tph-1(ot1274)
crh-1(tz2)
lin-29(ΔCRE)
ON
DIM
OFF
< 0.001
< 0.001
< 0.001
% of PHB expressing lin-29::SL2::GFP::H2B
22
18
24
20
Wild type
tph-1 (ot1274)
crh-1(tz2)
lin-29(ΔCRE)

Fig S2

Figure S2. LIN-29A expression in adult male PHB is controlled by sexual and cellular identity together with juvenile experience. Related to Figure 3.

(A) Quantification of the percentage of PHB neurons expressing *lin-29(xe63[gfp::lin-29a])* in wild-type well-fed males supplemented with octopamine (OA) or L1-starved males supplemented with serotonin (5-HT) during the starvation period.

(B) Schematic illustration of *lin-29(ot1597).* The *lin-29(ot1597)* allele is designed to delete the trunk of DNA elements, including the potential TRA-1 binding site at upstream ~11kb of the *lin-29a* locus in *lin-29(ot1482*). Representative images (middle) and quantification (bottom) of PHB neuron expressing *lin-29(ot1482[lin-29::SL2::GFP::H2B]* in wild type and *lin-29(ot1597)* in day 1 hermaphrodite.

(C,D) Longitudinal analysis of *lin-29(ot1482[lin-29::SL2::GFP::H2B])* expression in AVA neuron. Representative images (B) and quantification (C) of expression of AVA neuron expressing *lin-29(ot1482[lin-29::SL2::GFP::H2B])* in different time points after food exposure.

(E) Representative images (top) and quantification of (bottom) PHB neurons expressing *lin-29(ot1482[lin-29::SL2::GFP::H2B])* in day 1 adult males that undergo L1-starvation.

(F) Representative images (top) and quantification of (bottom) PHB neurons expressing *lin-29(ot1482[lin-29::SL2::GFP::H2B])* in wild type, *tph-1(ot1274)*, *crh-1(tz2)*, and *lin-29(ot1500)* day 1 adult males.

Statistics: *chi*-squared tests followed by Bonferroni multiple comparisons test. *p*-value and N numbers are indicated on the graph. The red dashed circle indicates PHB. Scale bar = 5 μm. + indicates the mean value.

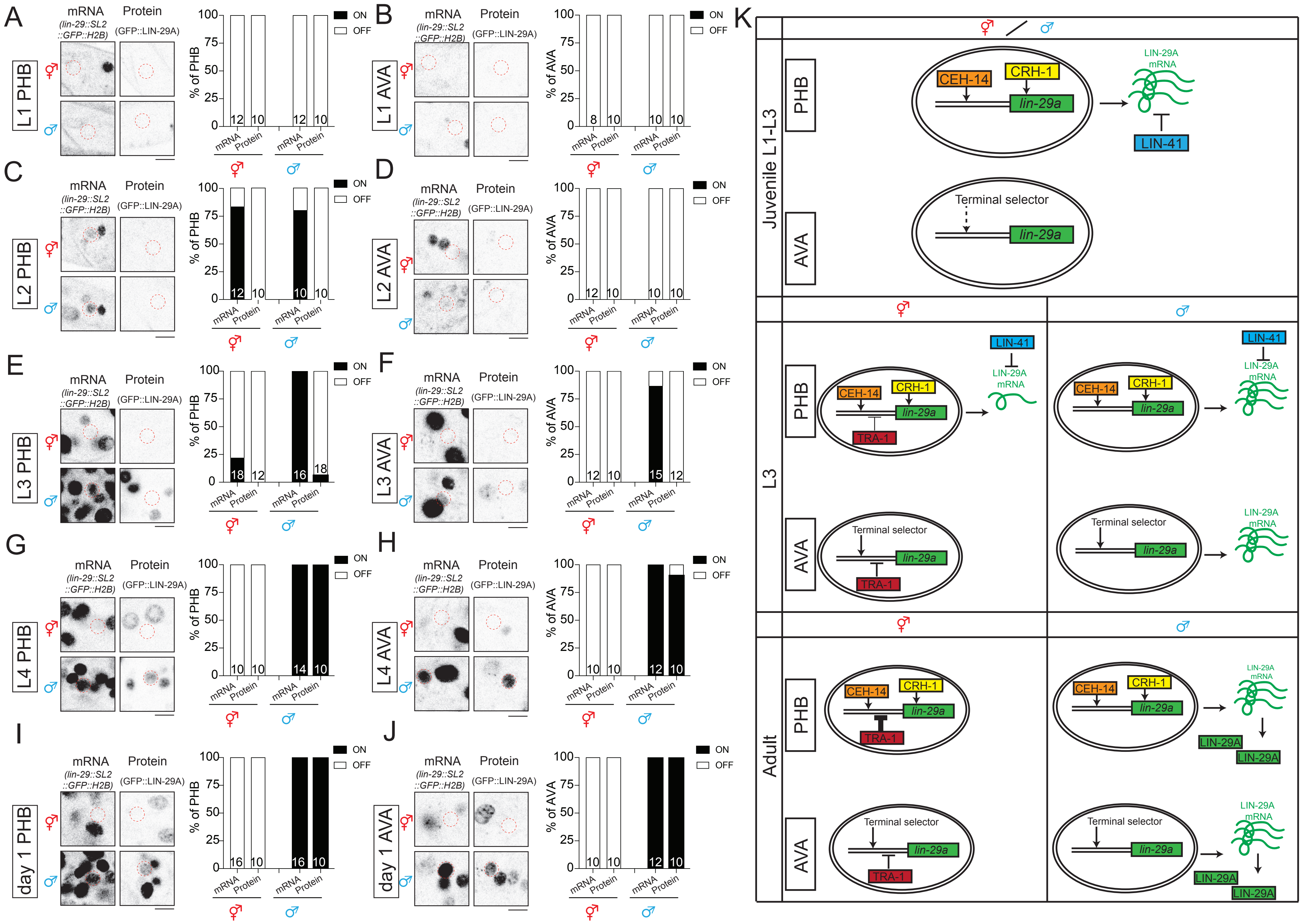

A
B
C
D
E
F
G
H
I
J
K
mRNA
Protein
(lin-29::SL2::GFP::H2B)
(GFP::LIN-29A)
L1 PHB
L1 AVA
L2 PHB
L2 AVA
L3 PHB
L3 AVA
L4 PHB
L4 AVA
day 1 PHB
day 1 AVA
% of PHB
% of AVA
ON
OFF
Juvenile L1-L3
L3
Adult
PHB
AVA
CEH-14
CRH-1
lin-29a
LIN-29A mRNA
LIN-41
Terminal selector
TRA-1
LIN-29A

Fig S3

Figure S3. Time course of *lin-29a* transcription and LIN-29A protein expression Related to Figure 4.

(A) Representative images (left) and quantification (right) of the percentage of PHB neurons expressing *lin-29(ot1482[lin-29::SL2::GFP::H2B])* and (*lin-29(xe63[gfp::lin-29a])* in wild-type well-fed hermaphrodites and males in L1 stage.

(B) Representative images (left) and quantification (right) of the percentage of AVA neurons expressing *lin-29(ot1482[lin-29::SL2::GFP::H2B])* and (*lin-29(xe63[gfp::lin-29a])* in wild-type well-fed hermaphrodites and males in L1 stage.

(C) Representative images (left) and quantification (right) of the percentage of PHB neurons expressing *lin-29(ot1482[lin-29::SL2::GFP::H2B])* and (*lin-29(xe63[gfp::lin-29a])* in wild-type well-fed hermaphrodites and males in L2 stage.

(D) Representative images (left) and quantification (right) of the percentage of AVA neurons expressing *lin-29(ot1482[lin-29::SL2::GFP::H2B])* and (*lin-29(xe63[gfp::lin-29a])* in wild-type well-fed hermaphrodites and males in L2 stage.

(E) Representative images (left) and quantification (right) of the percentage of PHB neurons expressing *lin-29(ot1482[lin-29::SL2::GFP::H2B])* and (*lin-29(xe63[gfp::lin-29a])* in wild-type well-fed hermaphrodites and males in L3 stage.

(F) Representative images (left) and quantification (right) of the percentage of AVA neurons expressing *lin-29(ot1482[lin-29::SL2::GFP::H2B])* and (*lin-29(xe63[gfp::lin-29a])* in wild-type well-fed hermaphrodites and males in L3 stage.

(G) Representative images (left) and quantification (right) of the percentage of PHB neurons expressing *lin-29(ot1482[lin-29::SL2::GFP::H2B])* and (*lin-29(xe63[gfp::lin-29a])* in wild-type well-fed hermaphrodites and males in L4 stage.

(H) Representative images (left) and quantification (right) of the percentage of AVA neurons expressing *lin-29(ot1482[lin-29::SL2::GFP::H2B])* and (*lin-29(xe63[gfp::lin-29a])* in wild-type well-fed hermaphrodites and males in L4 stage.

(I) Representative images (left) and quantification (right) of the percentage of PHB neurons expressing *lin-29(ot1482[lin-29::SL2::GFP::H2B])* and (*lin-29(xe63[gfp::lin-29a])* in wild-type well-fed hermaphrodites and males in day 1 stage.

(J) Representative images (left) and quantification (right) of the percentage of AVA neurons expressing *lin-29(ot1482[lin-29::SL2::GFP::H2B])* and (*lin-29(xe63[gfp::lin-29a])* in wild-type well-fed hermaphrodites and males in day 1 stage.

(K) Schematic illustration of the regulation of lin-29a transcription and translation during the development in PHB and AVA, respectively.

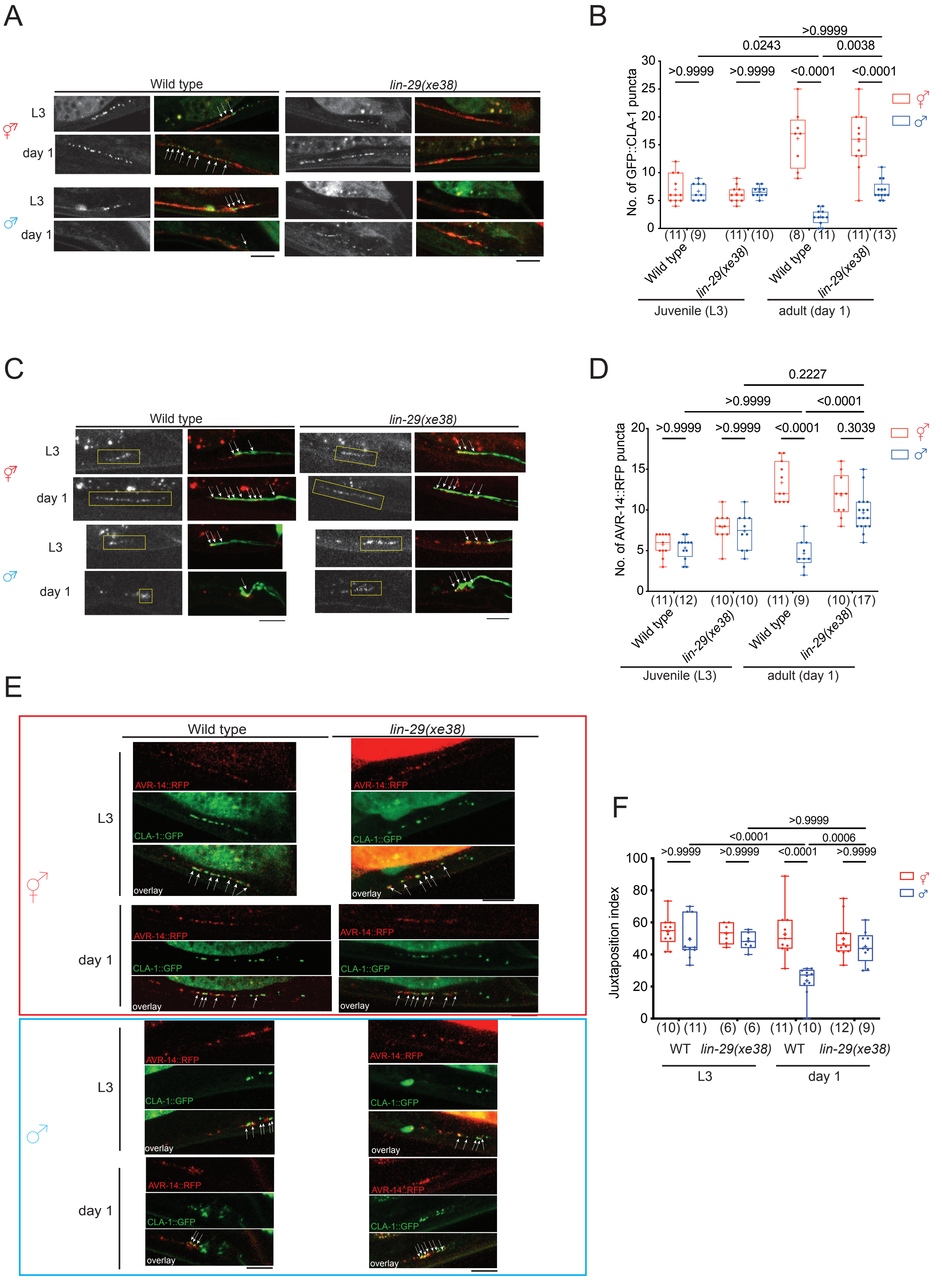

A
Wild type
lin-29(xe38)
L3
day 1
L3
day 1
B
>0.9999
0.0243
0.0038
>0.9999
>0.9999
<0.0001
<0.0001
No. of GFP::CLA-1 puncta
(11) (9) (11) (10) (8) (11) (11) (13)
Wild type
lin-29(xe38)
Wild type
lin-29(xe38)
Juvenile (L3)
adult (day 1)
C
Wild type
lin-29(xe38)
L3
day 1
L3
day 1
D
0.2227
>0.9999
<0.0001
>0.9999
>0.9999
<0.0001
0.3039
No. of AVR-14::RFP puncta
(11) (12) (10) (10) (11) (9) (10) (17)
Wild type
lin-29(xe38)
Wild type
lin-29(xe38)
Juvenile (L3)
adult (day 1)
E
Wild type
lin-29(xe38)
L3
day 1
AVR-14::RFP
CLA-1::GFP
overlay
F
>0.9999
<0.0001
0.0006
>0.9999
>0.9999
<0.0001
>0.9999
Juxtaposition index
(10) (11) (6) (6) (11) (10) (12) (9)
WT
lin-29(xe38)
WT
lin-29(xe38)
L3
day 1

Fig S4

Figure S4. Synapses visualized by juxtaposed PHB-localized CLA-1 and AVA-localized AVR-14 is affected by *lin-29a*. Related to Figure 5.

(A) Representative images of AVA-juxtaposed GFP::CLA-1 in PHB (*otIs883;otEx8040*) in L3 and day 1 wild-type and *lin-29(xe38)*.

(B) Quantification of AVA-juxtaposed GFP::CLA-1 in PHB(*otIs883; otEx8040*) in L3 and day 1 wild-type and *lin-29(xe38)*.

(C) Representative images of PHB-juxtaposed AVR-14::TagRFP (*otIs902*) in L3 and day 1 wild-type and *lin-29(xe38)*.

(D) Quantification of AVR-14::TagRFP in AVA (*otIs902; him-8(e1489)*) in L3 and day 1 wild-type and *lin-29(xe38)*.

(E) Representative images of the juxtaposition of PHB-localized GFP::CLA-1 and AVA-localized AVR-14::TagRFP (*otEx8163*) in L3 and day 1 wild-type and *lin-29(xe38)* hermaphrodites (top) and males (bottom).

(F) Quantification of juxtaposition index in in L3 and day 1 wild-type and *lin-29(xe38)* hermaphrodites and males.

Statistics: Two-way ANOVA followed by Bonferroni multiple comparisons test. *p*-value and N numbers are indicated on the graph. Scale bar = 10 µm. + indicates the mean value.

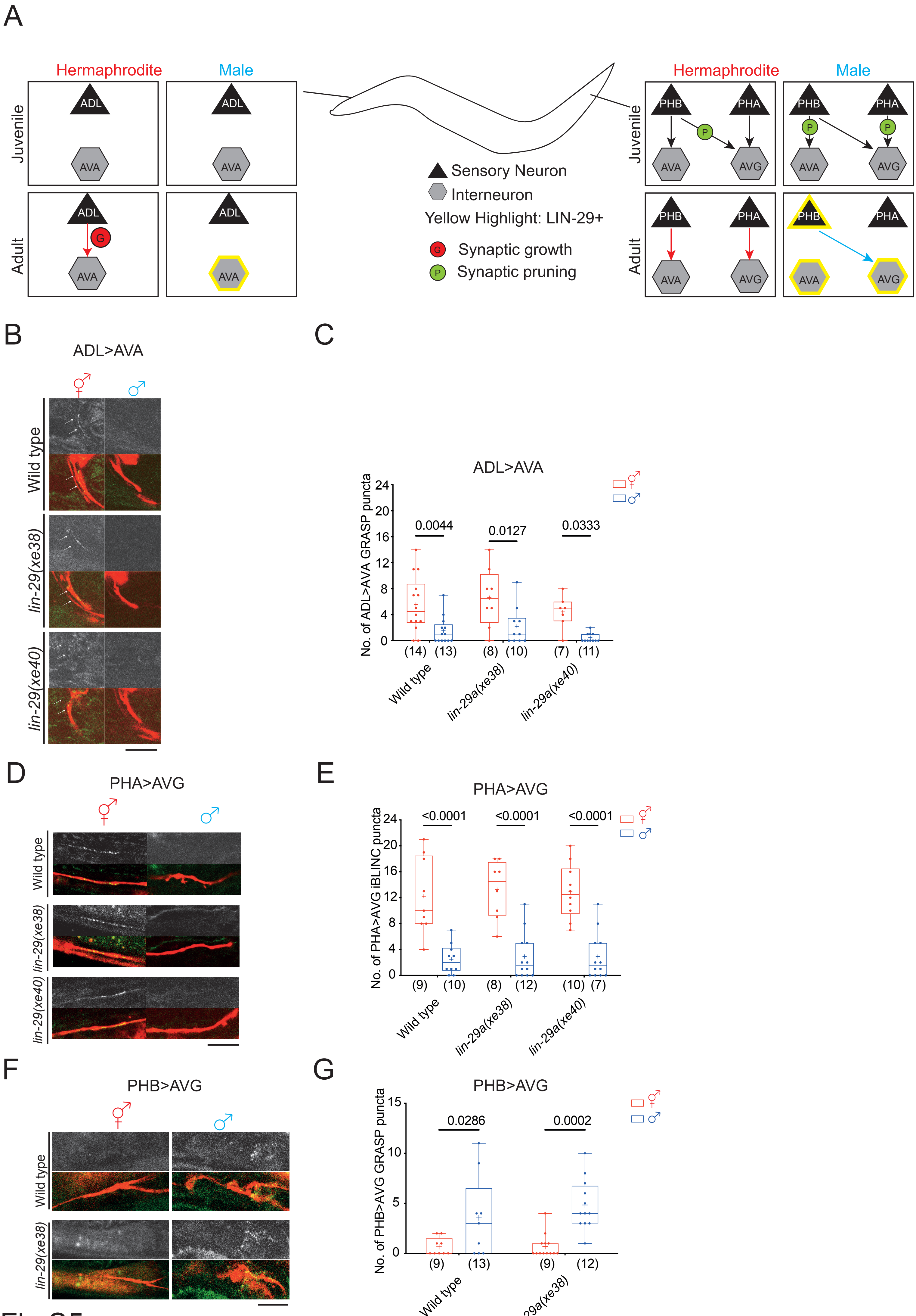

A
Hermaphrodite
Male
Juvenile
Adult
ADL
AVA
G
PHB
PHA
AVG
P
Sensory Neuron
Interneuron
Yellow Highlight: LIN-29+
Synaptic growth
Synaptic pruning
B
ADL>AVA
Wild type
lin-29(xe38)
lin-29(xe40)
C
ADL>AVA
No. of ADL>AVA GRASP puncta
0.0044
0.0127
0.0333
(14) (13) (8) (10) (7) (11)
Wild type
lin-29a(xe38)
lin-29a(xe40)
D
PHA>AVG
Wild type
lin-29(xe38)
lin-29(xe40)
E
PHA>AVG
No. of PHA>AVG iBLINC puncta
<0.0001
<0.0001
<0.0001
(9) (10) (8) (12) (10) (7)
Wild type
lin-29a(xe38)
lin-29a(xe40)
F
PHB>AVG
Wild type
lin-29(xe38)
G
PHB>AVG
No. of PHB>AVG GRASP puncta
0.0286
0.0002
(9) (13) (9) (12)
Wild type
lin-29a(xe38)

Fig S5

Figure S5. Loss of *lin-29a* does not affect other sexually dimorphic synapses. Related to Figure 5.

(A) Diagram of sexually dimorphic connections with neurons expressing LIN-29A upon sexual maturation.

(B,C) Representative images (B) and quantification (C)of ADL>AVA synaptic GRASP *(otEx6829)* in day 1 wild-type, *lin-29(xe38)* and *lin-29(xe40)* in both sexes.

(D,E) Representative images (D) and quantification (E) of PHA>AVG iBLINC (*otIs630*) in day 1 wild-type, *lin-29(xe38)* and *lin-29(xe40)* in both sexes.

(F,G) Representative images (F) and (G) quantification of PHB>AVG synaptic GRASP (*otIs614*) in day 1 wild-type and *lin-29(xe38)* in both sexes.

Statistics: (C,E,G)Two-way ANOVA followed by Bonferroni multiple comparisons test. *p*-value and N numbers are indicated on the graph. Scale bar = 10 μm. + indicates the mean value.

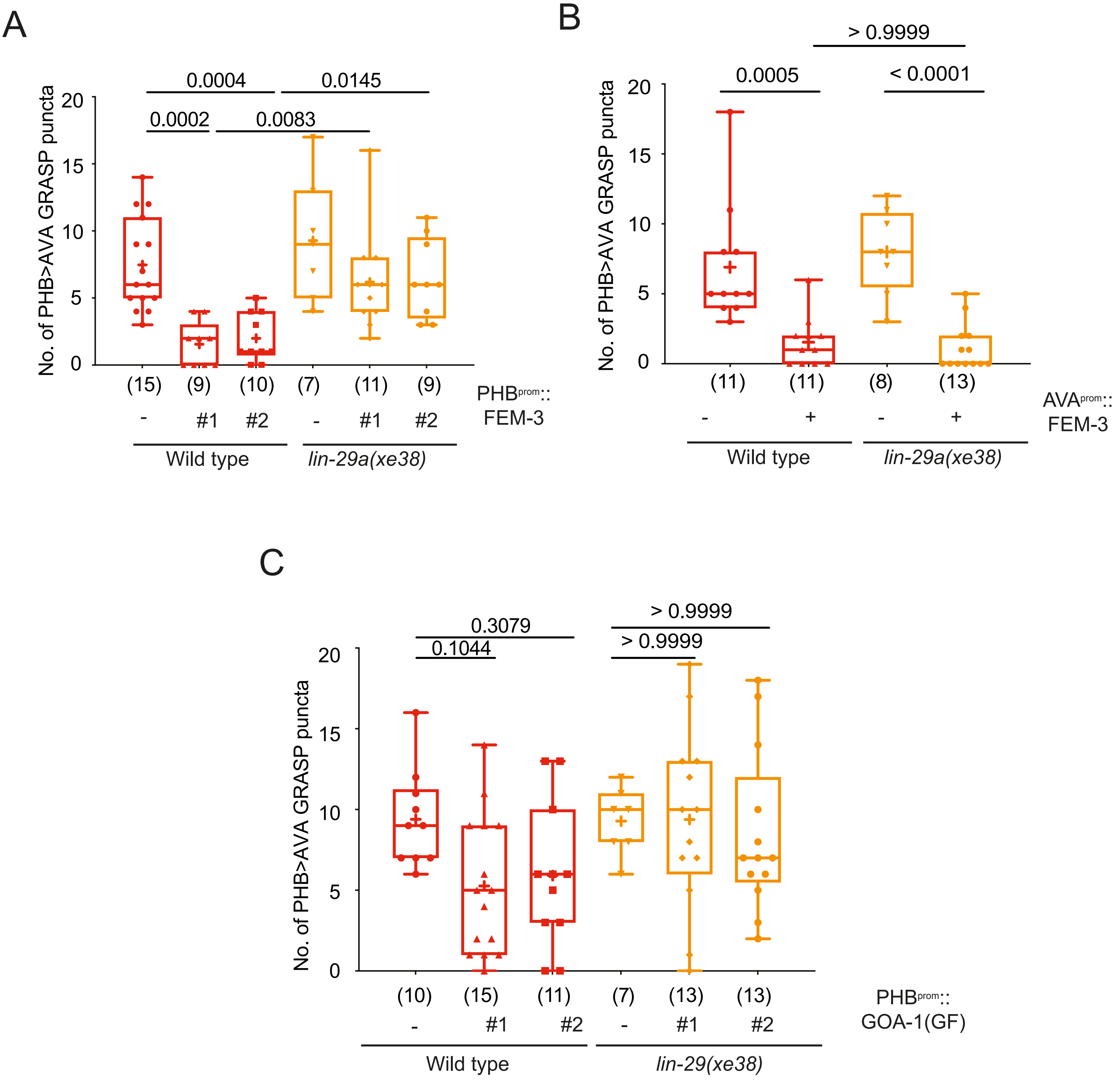

A
No. of PHB>AVA GRASP puncta
0.0004
0.0145
0.0002
0.0083
20
15
10
5
0
(15)
(9)
(10)
(7)
(11)
(9)
-
#1
#2
-
#1
#2
PHBprom::
FEM-3
Wild type
lin-29a(xe38)
B
No. of PHB>AVA GRASP puncta
> 0.9999
0.0005
< 0.0001
(11)
(11)
(8)
(13)
-
+
-
+
AVAprom::
FEM-3
Wild type
lin-29a(xe38)
C
No. of PHB>AVA GRASP puncta
0.3079
0.1044
> 0.9999
> 0.9999
(10)
(15)
(11)
(7)
(13)
(13)
-
#1
#2
-
#1
#2
PHBprom::
GOA-1(GF)
Wild type
lin-29(xe38)

Fig S6

Figure S6. Sexual identity acts through *lin-29a* in the PHB to control PHB>AVA sexual dimorphism. Related to Figure 5.

(A) Quantification of PHB>AVA synaptic GRASP(*otIs839*) in wild-type and *lin-29(xe38)* animals with transgene expressing PHB::FEM-3(*otEx7916* and *otEx8164*).

(B) Quantification of PHB>AVA synaptic GRASP(*otIs839*) in wild-type and *lin-29(xe38)* animals with transgene expressing AVA::FEM-3 (*otEx8165*).

(C) Quantification of PHB>AVA synaptic GRASP(*otIs839*) in wild-type and *lin-29(xe38)* animals with transgene expressing PHB::GOA-1^{gof}(*otEx7925* and *otEx8158*).

Statistics: One-way ANOVA followed by Bonferroni multiple comparisons test. *p*-value and N numbers are indicated on the graph. + indicates the mean value.

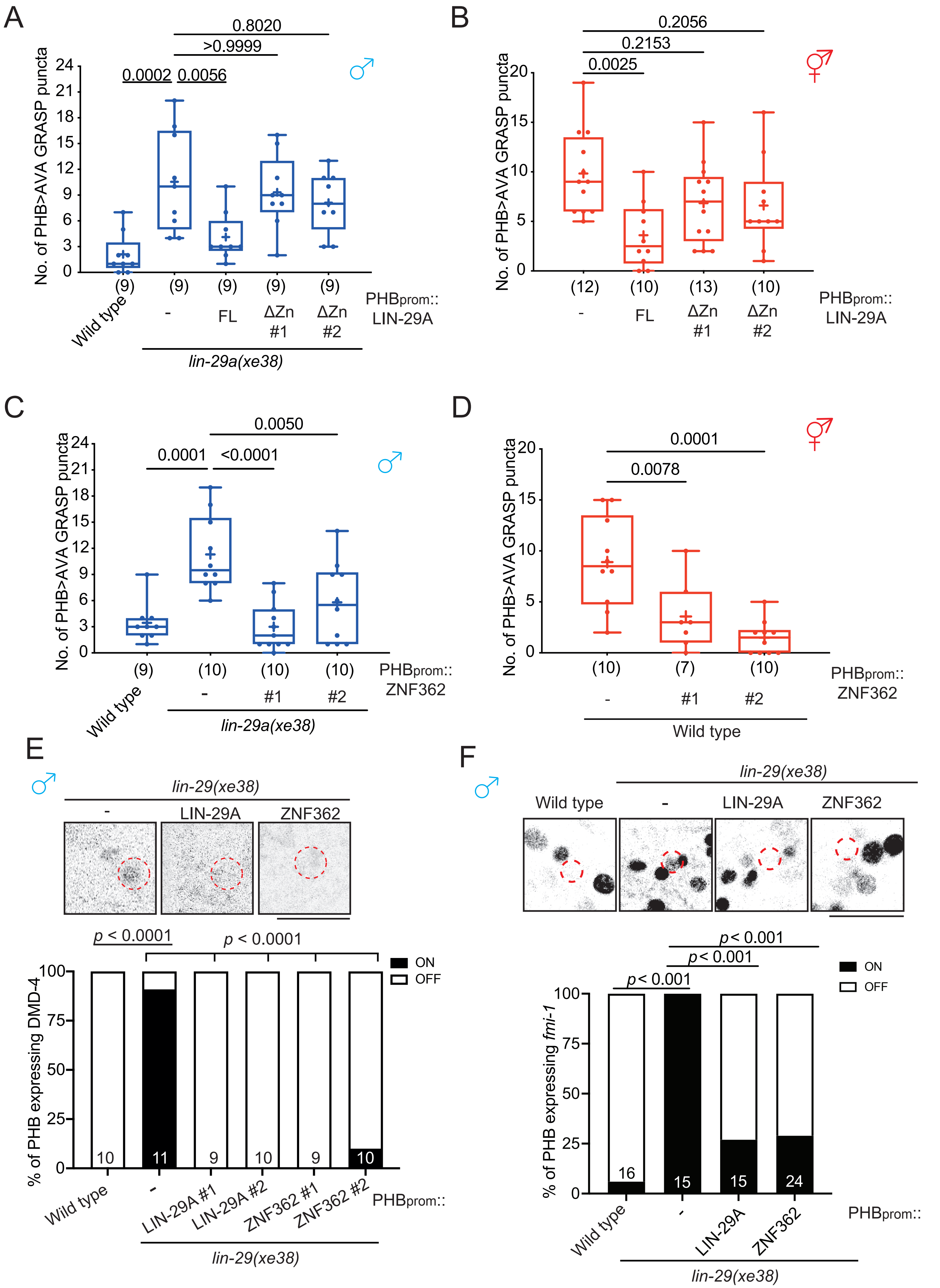
A
B
C
D
E
F
No. of PHB>AVA GRASP puncta
0.8020
>0.9999
0.0002
0.0056
Wild type
FL
ΔZn #1
ΔZn #2
PHBprom:: LIN-29A
lin-29a(xe38)
0.2056
0.2153
0.0025
0.0050
0.0001
<0.0001
0.0078
PHBprom:: ZNF362
Wild type
lin-29(xe38)
LIN-29A
ZNF362
p < 0.0001
% of PHB expressing DMD-4
ON
OFF
LIN-29A #1
LIN-29A #2
ZNF362 #1
ZNF362 #2
PHBprom::
p< 0.001
% of PHB expressing fmi-1

Fig S7

Figure S7. *lin-29a* mutants phenotypes are rescued by the human homolog of *lin29a*. Related to Figure 5,6,7.

(A,B) Quantification of PHB>AVA synaptic GRASP(*otIs839*) in and *lin-29(xe38)* males (A) and wild type hermaphrodites (B) with transgene expressing PHB::LIN-29$^{\Delta Zn}$(*otEx7928* and *otEx7929*).

(C,D) Quantification of PHB>AVA synaptic GRASP(*otIs839*) in and *lin-29(xe38)* males (A) and wild type hermaphrodites (B) with transgene expressing PHB::ZNF-362(o*tEx7930* and *otEx7931*).

(E) (top) Representative images and (bottom) quantification of PHB neuron expressing *dmd-4(ot935)* in *lin-29(xe38)* males with transgenes that express PHB::LIN-29A *(otEx7961 and otEx7964)* and PHB::ZNF362 *(otEx7997 and otEx7998)*

(F) Representative images (top) and quantification (bottom) of *fmi-1(syb4563)* expression in the PHB *lin-29(xe38)* males with transgenes that express PHB::LIN-29A *(otEx7961)* and PHB::ZNF362 *(otEx7997)*

(A,B,C,D) Two-way ANOVA and (E,F) Two-proportion Z test followed by Bonferroni multiple comparisons test. *p*-value and N numbers are indicated on the graph. Scale bar = 5 µm. + indicates the mean value.

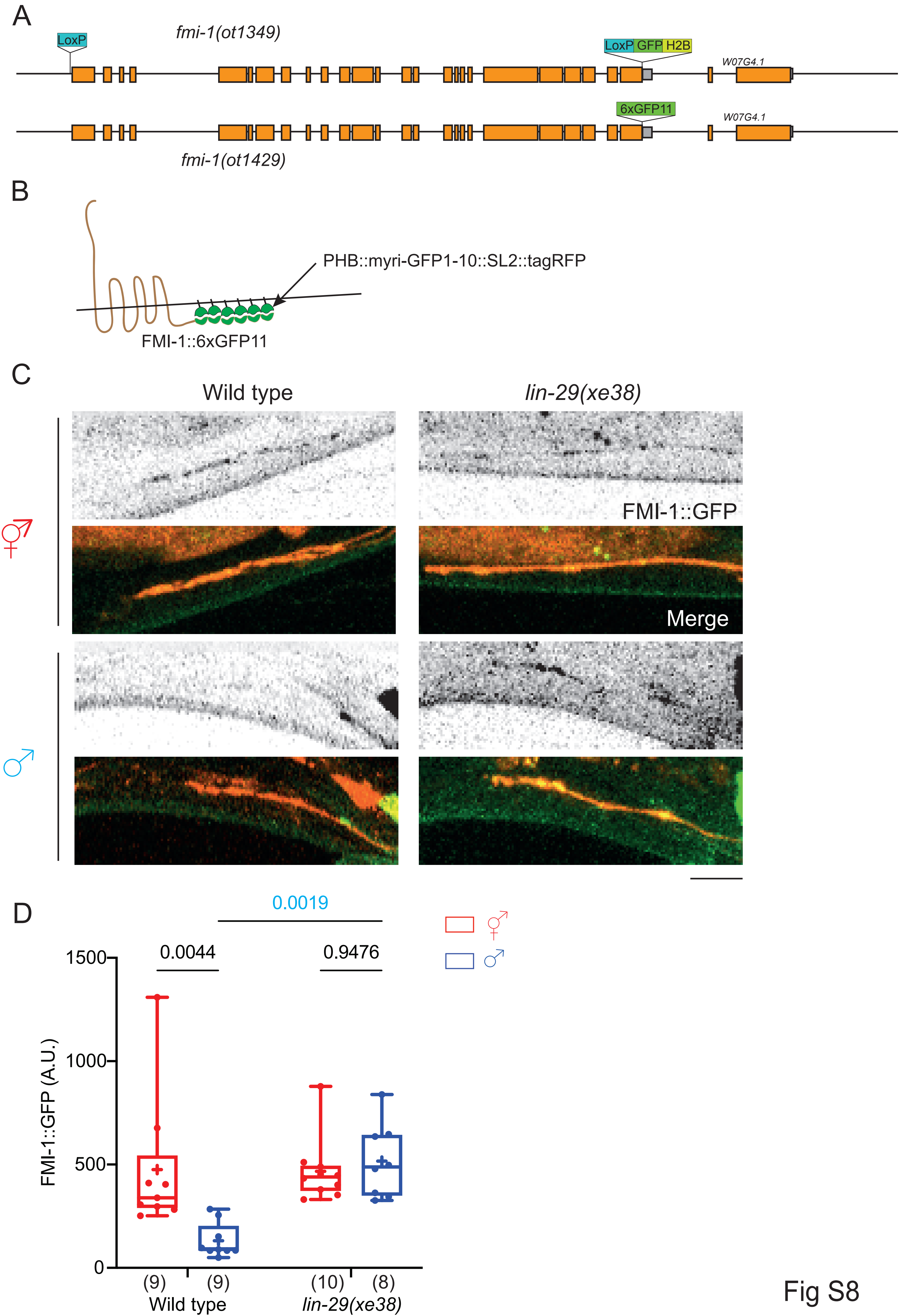
A
LoxP
fmi-1(ot1349)
LoxP GFP H2B
W07G4.1
6xGFP11
W07G4.1
fmi-1(ot1429)
B
PHB::myri-GFP1-10::SL2::tagRFP
FMI-1::6xGFP11
C
Wild type
lin-29(xe38)
FMI-1::GFP
Merge
D
0.0019
0.0044
0.9476
1500
1000
500
0
FMI-1::GFP (A.U.)
(9)
(9)
(10)
(8)
Wild type
lin-29(xe38)

Fig S8

Figure S8. Sexually dimorphic FMI-1 protein expression in the PHB axon is regulated by *lin-29a*. Related to Figure 7.

(A) Schematic illustration for *fmi-1(ot1349)* and *fmi-1(ot1429)*. *fmi-1(ot1349)* is a *fmi-1* allele in which the entire *fmi-1* locus is flanked with CRISPR/Cas9-engineered LoxP sites and a GFP::H2B tagged at the C-terminus region after second LoxP, after the *fmi-1* stop codon of *fmi-1*. Upon excision of *fmi-1* with Cre, the GFP::H2B is then expressed in the cell expressing Cre recombinase. *fmi-1(ot1429)* inserts codon-optimized 6XGFP$_{11}$ at the C-terminus.

(B) Schematic illustration of the visualization of the endogenous FMI-1 protein. The other half of GFP with myristoylation peptide (myri-GPF1-10) is expressed in the PHB by a transgene (*otEx8148*) to visualize the membrane-localized FMI-1.

(C,D) Representative images (C) and quantification (D) of endogenous FMI-1 expression from *fmi-1(ot1429); otEx8148* in day 1 wild type and *lin-29(xe38)* of both sexes.

Two-way ANOVA followed by Bonferroni multiple comparisons test. *p*-value and N numbers are indicated on the graph. Scale bar = 10 µm. + indicates the mean value.

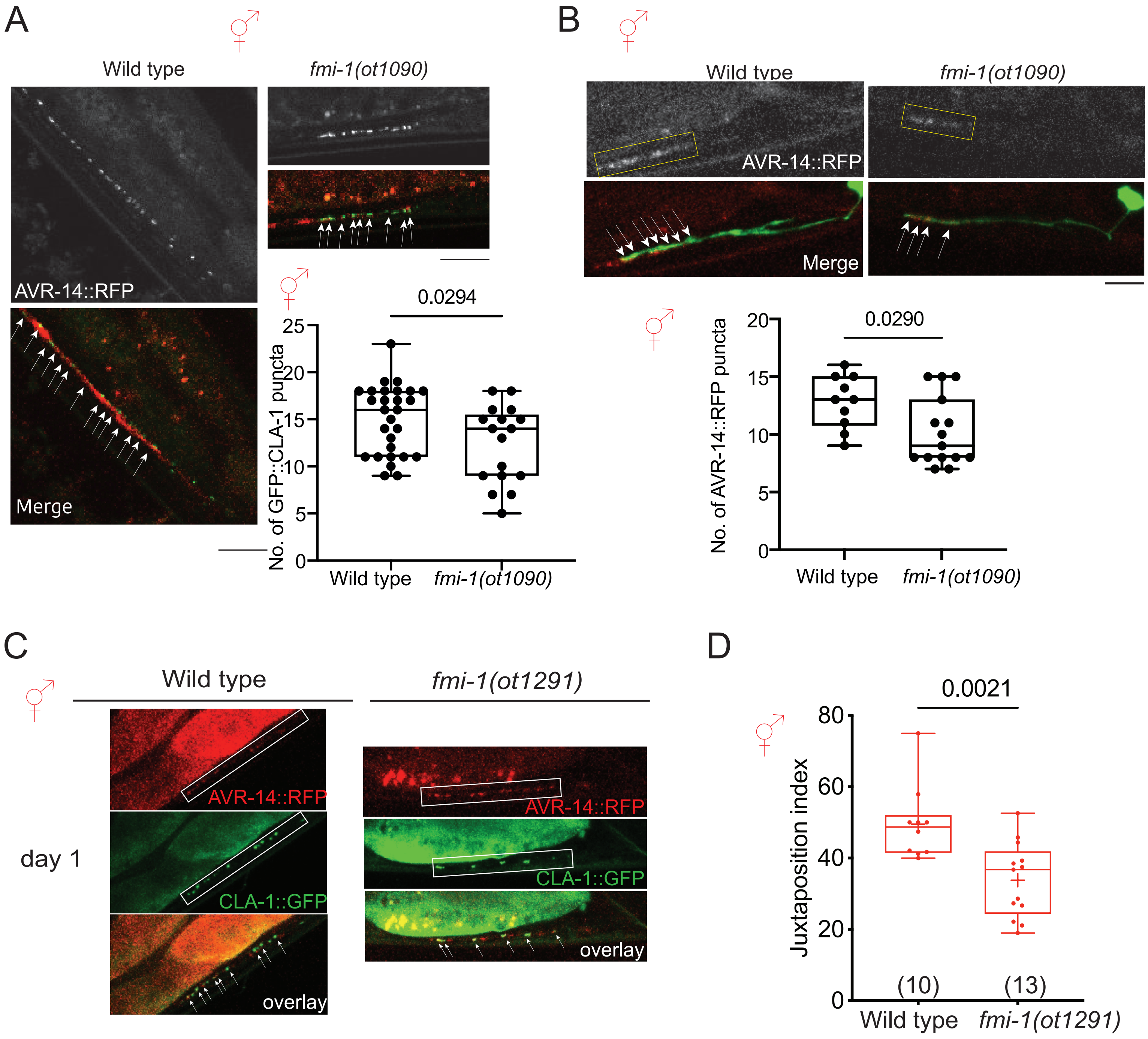

A
Wild type
fmi-1(ot1090)
AVR-14::RFP
Merge
0.0294
No. of GFP::CLA-1 puncta
25
20
15
10
5
0
Wild type
fmi-1(ot1090)
B
Wild type
fmi-1(ot1090)
AVR-14::RFP
Merge
0.0290
No. of AVR-14::RFP puncta
20
15
10
5
0
Wild type
fmi-1(ot1090)
C
Wild type
fmi-1(ot1291)
day 1
AVR-14::RFP
CLA-1::GFP
overlay
AVR-14::RFP
CLA-1::GFP
overlay
D
0.0021
Juxtaposition index
80
60
40
20
0
(10)
(13)
Wild type
fmi-1(ot1291)

Fig S9

Figure S9. Loss of *fmi-1* results in aberrant PHB>AVA connectivity. Related to Figure 8.

(A) Representative images (top) and quantification (bottom) of AVA-juxtaposed GFP::CLA-1 in PHB (*otIs883;otEx8040*) in day 1 wild-type and *fmi-1(ot1090)* hermaphrodites. *fmi-1(ot1090)* is also the *fmi-1* locus deletion, which removes all *fmi-1* isoforms, similar to that of *fmi-1(ot1291)*.

(B) Representative images (top) and quantification (D) of PHB-juxtaposed AVR-14::TagRFP (*otIs902*) in day 1 wild-type and *fmi-1(ot1090)* hermaphrodites.

(C) Representative images of the juxtaposition of PHB-localized GFP::CLA-1 and AVA-localized AVR-14::TagRFP (*otEx8163*) in day 1 wild-type and *fmi-1(ot1291)* hermaphrodites.

(D) Quantification of the juxtaposition of PHB-localized GFP::CLA-1 and AVA-localized AVR-14::TagRFP (*otEx8163*) in day 1 wild-type and *fmi-1(ot1291)* hermaphrodites.

(A,B,D) Student *t*-test and. *p*-value and N numbers are indicated on the graph. Scale bar = 10 µm. + indicates the mean value.

Table S1: Strain list

Genotype	Strain name	Source
lin-29(xe38)	HW1693	Pereira et al. 2019 *(21)*
lin-29(xe40)	HW1695	Pereira et al. 2019 *(21)*
lin-29(xe63)	HW2224	Pereira et al. 2019 *(21)*
lin-29(ot1396)	OH18669	This Study
lin-29(ot1482)	OH19114	This Study
lin-29(ot1500)	OH19177	This Study
lin-29(ot1597)	OH15580	This Study
tph-1(ot1274)	OH18147	This Study
crh-1(tz2)	YT17	Kimura et al., 2002 *(20)*
crh-1(ot1342)	OH18933	This Study
fmi-1(ot1090)	OH16772	This Study
fmi-1(ot1291)	OH18271	This Study
fmi-1(ot1349)	OH18441	This Study
fmi-1(ot1429)	OH18838	This Study
fmi-1(syb4563)	PHX4563	This Study
dmd-4(ot935)	OH15814	Bayer et al., 2020 *(12)*
dmd-4(ot957ot935)	OH15908	Bayer et al., 2020 *(12)*
lin-41(ma104)	CT8	Slack et al., 2000 *(30)*
lin-29(xe63) II; him-5(e1490) V; otEx8037[srab-20p:::goa-1^{gof}::SL2::TagRFP]	OH18905	This Study
tph-1(ot1274) lin-29(xe63) II; him-5(e1490) V;otEx8037[srab-20p::goa-1^{gof}::SL2::TagRFP]	OH18389	This Study
tph-1(ot1274) lin-29(xe63) II; crh-1(tz2) III; him-5(e1490) V;otEx8037[srab-20p::goa-1^{gof}::SL2::TagRFP]	OH18390	This Study
lin-29(xe63) II; crh-1(ot1342) III;him-5(e1490)/V; otIs839	OH18363	This Study
lin-29(xe63) II; crh-1(tz2) III; him-5(e1490) V; otEx8037[srab-20p::goa-1^{gof}::SL2::TagRFP]	OH18413	This Study
tph-1(ot1274) lin-29(xe63) II; him-5(e1490) V; otEx8053[srab-20p::crh-1^{WT}::SL2::TagRFP]	OH18439	This Study
tph-1(ot1274) lin-29(xe63) II; him-5(e1490) V;otEx8157[srab-20p::crh-1^{S48E}::SL2::TagRFP]	OH18906	This Study
tph-1(ot1274) lin-29(xe63) II; him-5(e1490) V; otEx8113[srab-20p::crh-1^{S48A}::SL2::TagRFP]	OH18636	This Study
lin-29(xe63) II; crh-1(tz2) III; him-5(e1490) V; otEx8053[srab-20p::crh-1^{WT}::SL2::TagRFP]	OH18907	This Study
lin-29(xe63) II; crh-1(tz2) III; him-5(e1490) V; otEx8157[srab-20p::crh-1^{S48E}::SL2::TagRFP]	OH18908	This Study
lin-29(xe63) II; crh-1(tz2) III; him-5(e1490) V; otEx8113[srab-20p::crh-1^{S48A}::SL2::TagRFP]	OH18909	This Study
lin-29(ot1396[ΔCRE]) II; him-5(e1490) V; otEx8119[AVA::TagRFP, PHB::TagRFP]	OH18669	This Study
lin-29(ot1482[lin-29::SL2::GFP::H2B]) II; him-5(e1490) V;otIs839	OH19115	This Study
tph-1(ot1274) lin-29(ot1482) II; him-5(e1490) V;otIs839	OH19168	This Study

lin-29(ot1482) II; crh-1(tz2) III;him-5(e1490) V;otIs839	OH19169	This Study
lin-29(ot1500[ΔCRE]ot1482) II; him-5(e1490) V;otIs839	OH19177	This Study
him-5(e1490) V;otIs839	OH17170	This Study
lin-29(xe38) II; him-5(e1490) V;otIs839;	OH18925	This Study
lin-29(xe40) II; him-5(e1490) V;otIs839;	OH18926	This Study
otIs883; otEx8040[flp-18p(AVA)::TagRFP]	OH18393	This Study
in-29(xe38) II; him-8(e1489) IV; otIs883; otEx8040[flp-18p(AVA)::TagRFP]	OH18394	This Study
him-8(e1489) IV; otIs902	OH18703	This Study
lin-29(xe38) II; him-8(e1489) IV; otIs902	OH18756	This Study
lin-29(xe38) II; him-5(1490) V; otIs839; otEx7763[flp-18p::lin-29a::SL2::3xNLSGFP]	OH17309	This Study
lin-29(xe38) II; him-5(e1490) V; otIs839; otEx7915[srab-20p::lin-29a::SL2::3xNLS::GFP]	OH17828	This Study
lin-29(xe38) II; him-5(1490) V; otIs839; otEx7790[gpa-6p::lin-29a::SL2::3XNLS::GFP]	OH18927	This Study
him-5(1490) V; otIs839; otEx7790[gpa-6p::lin-29a::SL2::3XNLS::GFP]	OH17411	This Study
him-5(1490) V; otIs839; otEx7763[flp-18p::lin-29a::SL2::3xNLSGFP]	OH18928	This Study
him-5(e1490) V; otIs839; otEx7915[srab-20p::lin-29a::SL2::3xNLS::GFP]	OH18929	This Study
him-5(e1490) V; otIs839; otEx7925[srab-20p::goa-1^{gof}::SL::3xNLS::GFP]	OH17867	This Study
him-5(e1490) V; otIs839; otEx8158[srab-20p::goa-1^{gof}::SL::3xNLS::GFP]	OH18911	This Study
lin-29(xe38) II; him-5(e1490) V; otIs839; otEx7925[Psrab-20::goa-1(gof)::SL::3xNLS::GFP]	OH18930	This Study
lin-29(xe38) II; him-5(e1490) V; otIs839; otEx8158[srab-20p::goa-1^{gof}::SL::3xNLS::GFP]	OH18931	This Study
tph-1(ot1274) II; him-5(e1490) V; otIs839	OH18147	This Study
tph-1(ot1274) lin-29(xe38) II; him-5(e1490) V; otIs839	OH18932	This Study
crh-1(tz2) III ; him-5(e1490) V; otIs839	OH18217	This Study
crh-1(ot1342) III; him-5(e1490) V; otIs839	OH18933	This Study
tph-1(ot1274) II; him-5(e1490) V; otIs839; otEx7925[srab-20p::goa-1^{gof}:SL::3xNLS::GFP]	OH18934	This Study
tph-1(ot1274) II; him-5(e1490) V; otIs839; otEx7915[srab-20p::lin-29a::SL2::3xNLS::GFP]	OH18935	This Study
tph-1(ot1274) lin-29(xe38) II; him-5(e1490) V; otIs839; otEx7925[srab-20p::goa-1^{gof}::SL::3xNLS::GFP]	OH18936	This Study
tph-1(ot1274) lin-29(xe38) II; him-5(e1490) V; otIs839; otEx7915[srab-20p::lin-29a::SL2::3xNLS::GFP]	OH18937	This Study
tph-1(ot1274) II; otIs839; him-5(e1490) V; otEx8045[srab-20p::crh-1^{WT}::SL2::3xNLS::GFP]	OH18415	This Study
tph-1(ot1274) II; otIs839; him-5(e1490) V; otEx8046[srab-20p::crh-1^{S48E}::SL2::3xNLS::GFP]	OH18416	This Study
tph-1(ot1274) II; otIs839; him-5(e1490) V; otEx8082[srab-20p::crh-1^{S48A}::SL2::3xNLS::GFP]	OH18504	This Study

crh-1(tz2) III; otIs839; him-5(e1490) V; otEx8045[srab-20p::crh-1^{WT}::SL2::3xNLS::GFP]	OH18912	This Study
crh-1(tz2)III; otIs839; him-5(e1490) V; otEx8046[srab-20p::crh-1^{S48E}::SL2::3xNLS::GFP]	OH18913	This Study
crh-1(tz2) III; otIs839; him-5(e1490) V; otEx8082[srab-20p::crh-1^{S48A}::SL2::3xNLS::GFP]	OH18914	This Study
him-5(e1490) V;dmd-4(ot935) X; otIs839	OH18938	This Study
lin-29(xe38) II; him-5(e1490) V;dmd-4(ot935) X; otIs839	OH18939	This Study
tph-1(ot1274) II; him-5(e1490) V;dmd-4(ot935) X; otIs839	OH18372	This Study
crh-1(ot1342) III; him-5(e1490) V;dmd-4(ot935) X; otIs839	OH18940	This Study
him-5(e1490) V; dmd-4(ot957ot935) X; otIs839	OH18084	This Study
lin-29(xe38) II; him-5(e1490) V; dmd-4(ot957ot935) X; otIs839	OH18085	This Study
lin-29(xe38) II; him-5(e1490) V; dmd-4(ot935) X;otEx7961[srab-20p::lin-29a::SL2::TagRFP]	OH18094	This Study
lin-29(xe38) II; him-5(e1490) V; dmd-4(ot935) X; otEx7964[srab-20p::lin-29a::SL2::TagRFP]	OH18097	This Study
lin-29(xe38) II; him-5(e1490) V; dmd-4(ot935) X; otEx7997[srab-20p::znf-362::SL2::TagRFP]	OH18228	This Study
lin-29(xe38) II; him-5(e1490) V; dmd-4(ot935) X;otEx7998[srab-20p::znf-362::SL2::TagRFP]	OH18231	This Study
lin-29(xe38) II; him-5(e1490) V; dmd-4(ot935) X; otEx8159[srg-13p::lin-29a::SL2::TagRFP]	OH18915	This Study
lin-29(xe38)II; him-5(e1490) V; dmd-4(ot935) X; otEx8160[srg-13p::lin-29a::SL2::TagRFP]	OH18916	This Study
lin-29(xe38) II; him-5(1490) V; otIs839; otEx7984[gpa-6p::dmd-4::GFP]	OH18186	This Study
lin-29(xe38) II; him-5(1490) V; otIs839; otEx7983[srg-13p::dmd-4::GFP]	OH18185	This Study
him-5(1490) V; otIs839; otEx7984[gpa-6p::dmd-4::GFP]	OH18941	This Study
otIs839; otEx7983[srg-13p::dmd-4::GFP]	OH18942	This Study
him-8(e1489) IV; fmi-1(syb4563) V; otIs839	OH18943	This Study
lin-29(xe38) II; him-8(e1489) IV; fmi-1(syb4563) V; otIs839	OH18924	This Study
him-8(e1489) IV; fmi-1(syb4563) V; dmd-4(ot957ot935) X; otIs839	OH18272	This Study
lin-29(xe38) II; him-8(e1489) IV; fmi-1(syb4563) V; dmd-4(ot957ot935) X; otIs839	OH18982	This Study
him-8(e1489) IV; fmi-1(syb4563) V; otEx7961[srab-20p::lin-29a::SL2::TagRFP]	OH18944	This Study
tph-1(ot1274) II; him-8(e1489) IV; fmi-1(syb4563) V; otIs839	OH18945	This Study
crh-1(ot1342) III; him-8(e1489) IV; fmi-1(syb4563) V; otIs839	OH18946	This Study

lin-29(xe38) II; him-8(e1489) IV; fmi-1(syb4563) V; otEx7961[srab-20p::lin-29a::SL2::TagRFP]	OH18985	This Study
him-8 (e1489) IV; fmi-1(syb4563) V; otEx8083[srab-20p::dmd-4::SL2::TagRFP]	OH18505	This Study
him-8(e1489) IV; fmi-1(ot1291) V; otIs839	OH18271	This Study
lin-29(xe38) II; him-8(e1489) IV; fmi-1(ot1291) V;otIs839	OH18387	This Study
otIs839;him-5(e1490) V; otEx8032[srab-20p::fmi-1a::SL2::3xNLS::GFP]	OH18370	This Study
otIs839;him-5(e1490) V; otEx8033[srab-20p::fmi-1a::SL2::3xNLS::GFP]	OH18371	This Study
him-8(e1489) IV; fmi-1(ot1291) V; otIs839; otEx8032[srab-20p::fmi-1a::SL2::3xNLS::GFP]	OH19030	This Study
him-8(e1489) IV; fmi-1(ot1291) V; otIs839; otEx8033[srab-20p::fmi-1a::SL2::3xNLS::GFP]	OH19031	This Study
otIs839; him-5(e1490) fmi-1(ot1349) V	OH18441	This Study
otIs839; him-5(e1490) fmi-1(ot1349) V; otEx8062[srab-20p::3xNLS::Cre]	OH18461	This Study
otIs839; him-5(e1490) fmi-1(ot1349) V; otEx8063[UPN::3xNLS::Cre]	OH18467	This Study
otIs839; him-5(1490)fmi-1(ot1349)V;otEx8064[flp-18p::3xNLS::Cre]	OH18468	This Study
otIs839; him-5(e1490) fmi-1(ot1349)V;otEx8084[hsp-16.2p::3xNLS::Cre]	OH18506	This Study
otIs839; him-5(e1490) fmi-1(ot1349)V;otEx8161[srab-20p::3xNLS::Cre]	OH18917	This Study
otIs839; him-5(1490)fmi-1(ot1349)V;otEx8162[flp-18p::3xNLS::Cre]	OH18918	This Study
otIs839; him-5(e1490) fmi-1(ot1349)V;otEx8084[hsp-16.2p::3xNLS::Cre]	OH18506	This Study
him-8(e1489) IV;otEx8152[srab-20p::TagRFP, srab-20p::CD4::GFP$_{1-10}$,flp-18p::TagRFP, flp-18::CD4::GFP$_{11}$]	OH18858	This Study
him-8(e1489) IV; fmi-1(ot1291) V;otEx8152[srab-20p::TagRFP, srab-20p::CD4::GFP$_{1-10}$,flp-18p::TagRFP, flp-18::CD4::GFP$_{11}$]	OH18860	This Study
him-8(e1489) IV; fmi-1(ot1291) V; otEx8152[srab-20p::TagRFP, srab-20p::CD4::GFP$_{1-10}$,flp-18p::TagRFP, flp-18::CD4::GFP$_{11}$], otEx8032[srab-20p::fmi-1a::SL2::3xNLS::GFP]	OH18979	This Study
him-5(e1490) IV; fmi-1(ot1349)V; otEx8084[hsp-16.2p::3xNLS::Cre::p10UTR],otEx8152[srab-20p::TagRFP, srab-20p::CD4::GFP$_{1-10}$,flp-18p::TagRFP, flp-18::CD4::GFP$_{11}$]	OH19023	This Study
lin-29(xe38) II; otIs839; him-5(e1490) V; otEx7916[gpa-6p::fem-3::SL2::2xNLS::TagRFP-T]	OH17830	This Study
otEx6829	OH14590	Majeed et al., 2024 (*48)*
lin-29(xe38) II; otIs614; him-5(e1490) V	OH18553	This Study

otIs614	OH13577	Oren-Suissa et al., 2016 *(6)*
otIs630	OH14099	Oren-Suissa et al., 2016 *(6)*
him-8(e1489) IV; otEx8176[srab-20p::gfp::cla-1 15ng/ul, flp-18p::avr-14::TagRFP]	OH18983	This Study
him-8(e1489) IV; fmi-1(ot1291) V;otEx8176[srab-20p::gfp::cla-1 15ng/ul, flp-18p::avr-14::TagRFP]	OH18984	This Study
lin-29(xe38) II; him-8(e1489) IV; otEx8176[srab-20p::gfp::cla-1 15ng/ul, flp-18p::avr-14::TagRFP]	OH19008	This Study
lin-29(xe38) II; otIs839;him-5(e1490) V; otEx7916[gpa-6p::fem-3::SL2::2xNLS::TagRFP-T]	OH17830	This Study
otIs839; him-5(e1490) V; otEx8164[gpa-6p::fem-3::SL2::2xNLS::TagRFP-T]	OH18920	This Study
otIs839; him-5(e1490) V; otEx8165[flp-18p::fem-3::SL2::2xNLS::TagRFP-T]	OH18921	This Study
lin-29(xe38) II;otI839; him-5(e1490) V; otEx7929[srab-20p::lin-29a(delZn)::SL2::3xNLS::GFP]	OH17984	This Study
otIs839; him-5(e1490) V; otEx7928[srab-20p::lin-29a(delZn)::SL2::3xNLS::GFP]	OH17881	This Study
lin-29(xe38) II;otI839; him-5(e1490) V; otEx7929[srab-20p::lin-29a(delZn)::SL2::3xNLS::GFP]	OH17984	This Study
otIs839; him-5(e1490) V; otEx7930[srab-20p::znf362::SL2::3xNLS::GFP]	OH17895	This Study
otIs839; him-5(e1490) V; otEx7931[srab-20p::znf362::SL2::3xNLS::GFP]	OH17896	This Study
lin-29(xe38) II;otI839; him-5(e1490) V; otEx7929[srab-20p::lin-29a(delZn)::SL2::3xNLS::GFP]	OH17984	This Study
lin-29(xe38) II; him-5(e1490) V; dmd-4(ot935)/X; otEx7997[srab-20p::znf-362::SL2::TagRFP]	OH18228	This Study
lin-29(xe38) II; him-5(e1490) V; dmd-4(ot935)/X;otEx7998[srab-20p::znf-362::SL2::TagRFP]	OH18231	This Study
tph-1(ot1274) II;; him-8(e1489) IV; otIs902	OH18769	This Study
him-5(e1490) fmi-1(ot1429)[fmi-1::6xGFP11] V	OH18838	This Study
him-5(e1490) fmi-1(ot1429)[fmi-1::6xGFP11] V;otEx8148[srab-20p::myriGFP::SL2::TagRFP]	OH18839	This Study
lin-29(xe38) II; him-5(e1490) fmi-1(ot1429)[fmi-1::6xGFP11] V;otEx8148[srab-20p::myriGFP::SL2::TagRFP]	OH18887	This Study
otIs839; him-5(e1490) fmi-1(ot1349) V;otEx8084[Phsp-16.2::3xNLS::Cre]	OH18506	This Study
lin-29(xe38) II;fmi-1(ot1349) him-5(e1490) V; otIs839; otEx8084[hsp-16.2p::3xNLS::Cre]	OH19231	This Study
lin-29(ot1597) II; him-5 V; otIs839	OH19580	This Study
